# Eight-week multi-domain cognitive training does not impact large-scale resting-state brain networks in Parkinson’s disease

**DOI:** 10.1016/j.nicl.2022.102952

**Published:** 2022-01-30

**Authors:** Tim D. van Balkom, Odile A. van den Heuvel, Henk W. Berendse, Ysbrand D. van der Werf, Chris Vriend

**Affiliations:** a Amsterdam UMC, Vrije Universiteit Amsterdam, Psychiatry, Amsterdam Neuroscience, De Boelelaan 1117, Amsterdam, Netherlands; b Amsterdam UMC, Vrije Universiteit Amsterdam, Anatomy and Neurosciences, Amsterdam Neuroscience, De Boelelaan 1117, Amsterdam, Netherlands; c Amsterdam UMC, Vrije Universiteit Amsterdam, Neurology, Amsterdam Neuroscience, De Boelelaan 1117, Amsterdam, Netherlands

**Keywords:** Parkinson’s disease, Cognitive training, Cognitive function, Resting-state fMRI, Graph theory, Functional connectivity, AC, Active control, BC, Betweenness centrality, CC, Clustering coefficient, COGTIPS, COGnitive Training In Parkinson Study, CT, Cognitive training, dACC, Dorsal anterior cingulate cortex, DMN, Default mode network, dlPFC, Dorsolateral prefrontal cortex, FPN, Frontoparietal network, GE, Global efficiency, PC, Participation coefficient, PD, Parkinson’s disease, PD-D, PD dementia, PD-MCI, PD mild cognitive impairment, PD-NC, PD normal cognition, Q, Modularity, Rs-fMRI, Resting-state functional MRI, SCWT, Stroop Color-Word Test, SN, Salience network, ToL, Tower of London, WM, white matter

## Abstract

There is *meta*-analytic evidence for the efficacy of cognitive training (CT) in Parkinson’s disease (PD). We performed a randomized controlled trial where we found small positive effects of CT on executive function and processing speed in individuals with PD (n_total_ = 140). In this study, we assessed the effects of CT on brain network connectivity and topology in a subsample of the full study population (n_mri_ = 86). Participants were randomized into an online multi-domain CT and an active control condition and performed 24 sessions of either intervention in eight weeks. Resting-state functional MRI scans were acquired in addition to extensive clinical and neuropsychological assessments pre- and post-intervention. In line with our preregistered analysis plan (osf.io/3st82), we computed connectivity between ‘cognitive’ resting-state networks and computed topological outcomes at the whole-brain and sub-network level. We assessed group differences after the intervention with mixed-model analyses adjusting for baseline performance and analyzed the association between network and cognitive performance changes with repeated measures correlation analyses. The final analysis sample consisted of 71 participants (n CT = 37). After intervention there were no group differences on between-network connectivity and network topological outcomes. No associations between neural network and neuropsychological performance change were found. CT increased segregated network topology in a small sub-sample of cognitively intact participants. Post-hoc nodal analyses showed post-intervention enhanced connectivity of both the dorsal anterior cingulate cortex and dorsolateral prefrontal cortex in the CT group. The results suggest no large-scale brain network effects of eight-week computerized CT, but rather localized connectivity changes of key regions in cognitive function, that potentially reflect the specific effects of the intervention.

## Highlights

• Cognitive training does not enhance resting-state brain networks in Parkinson’s disease.
• Effects of the intervention rather may target relevant prefrontal brain connections.
• Future research should focus on cognitive training in specific cognitive subgroups.

## 1 Introduction

Cognitive impairment is a common and debilitating non-motor symptom of Parkinson’s disease (PD; Aarsland et al., 2017, Svenningsson et al., 2012), which is already present at diagnosis in a quarter of PD patients and can ultimately lead to PD dementia in the large majority of patients (Aarsland et al., 2017, Muslimovic et al., 2005). In late stages of PD, rivastigmine is moderately effective in relieving cognitive impairment related to PD dementia (Meng et al., 2019, Noufi et al., 2019, Seppi et al., 2011). To date, there is little evidence for efficacy of treatment of cognitive impairment pre-dementia, although cognitive training (CT) has shown promising results (Leung et al., 2015). CT is a relatively cost-efficient and easy-to-administer therapy option without the side-effects that are often caused by adjuvant medication (Seppi et al., 2011). In an earlier report, we showed that eight-week online, multi-domain CT had small positive effects on executive function and processing speed in a large sample of PD patients (of which current study describes a subsample; van Balkom et al., 2021 (preprint)). These effects were accompanied by white matter microstructure alterations suggesting a higher incidence of crossing fibers in the anterior limb of the internal capsule connecting subcortical structures (Vriend et al., 2021).

While cognitive impairment in PD is associated with widespread cortical atrophy (Laansma et al., 2020, Pereira et al., 2014), altered structural and functional connectivity (Fiorenzato et al., 2019, Galantucci et al., 2017, Gorges et al., 2019, Olde Dubbelink et al., 2014b, Trujillo et al., 2015b), and disrupted brain networks (Baggio et al., 2015, Putcha et al., 2015, Putcha et al., 2016, Wolters et al., 2019), to date little is known about the impact of CT on these neural alterations. CT is thought to induce neuroplastic effects by repeated (challenging) cognitive engagement and there is already a plethora of studies that have shown effects of cognitive training on brain morphometry, activity and connectivity. In a review on the neural correlates of CT we describe how CT counteracts dysfunctional brain network changes that are associated with aging and neurodegenerative processes by enhancing compensatory mechanisms (such as increased neural activity during task performance) and normalizing functional connectivity (van Balkom et al., 2020). Specifically, CT seemed to specifically target the configuration of intrinsic functional brain networks, i.e. the frontoparietal network (FPN), salience network (SN) and default mode network (DMN), whose interactions play an important role in mediating cognitive function (Menon, 2011, Menon and Uddin, 2010, Spreng et al., 2009). Nevertheless, only one study in PD patients was included in this review (Díez-Cirarda et al., 2016). Two earlier, exploratory studies on CT in PD that were not included in this review because of the small sample sizes showed decreased regional brain activity during executive function (Nombela et al., 2011) and increased intrinsic functional activity (i.e., during resting-state) in regions of attention-related and frontoparietal resting-state networks (Cerasa et al., 2014).

Rather than focusing on morphometry or connectivity of a single brain area, contemporary neuroimaging methods are used to study the brain as a complex network: a ‘graph’. Complex networks have distinctive properties, such as small-world organization (i.e., an integrated but simultaneously clustered network), a modular structure, and a power-law degree distribution (i.e., only a small proportion of network nodes has many connections; Kim and Wilhelm, 2008, Rubinov and Sporns, 2010, Stam and Reijneveld, 2007). Inter-individual differences in the topology of the human neural network are associated with differences in cognitive and emotional function (Medaglia et al., 2015). Individuals with PD show abnormalities in functional and structural network topology such as a progressive decline in local and global efficiency (Luo et al., 2015, Olde Dubbelink et al., 2014a, Pereira et al., 2015), decreased clustering (Luo et al., 2015, Olde Dubbelink et al., 2014a, Vriend et al., 2018), and reorganization of highly connected regions (‘hubs’; Baggio et al., 2014, Pereira et al., 2015), but also increased modularity and local efficiency which is presumed to be compensatory (Baggio et al., 2014). How CT may normalize the topology of the neural network is largely unknown and not studied in PD. In healthy young subjects, working memory training increased modularity (Finc et al., 2020), small-world organization (Langer et al., 2013), and modular efficiency and node strength (Roman et al., 2017), while cognitive strategy training decreased modularity in individuals with traumatic brain injury (Han et al., 2020). Modularity may be of particular interest as it has repeatedly been recognized as a predictor of therapeutic efficacy (Arnemann et al., 2015, Baniqued et al., 2017, Gallen et al., 2016).

In this study, we elaborated on the clinical findings reported elsewhere (van Balkom et al., 2021), showing small positive effects of CT on speed of processing during executive function tasks. We assessed the effect of eight-week online multi-domain CT relative to an active control condition on neural network connectivity and topology in a large sample of individuals with PD. We investigated the effect of CT on whole-brain network properties using graph indices and also assessed the effect on the FPN, SN and DMN. We hypothesized that, on a global level, CT increases neural efficiency, modularity and participation coefficient (i.e., ratio between connections within and between modules). For the intrinsic functional networks, we hypothesized that 1) CT increases segregation of neurocognitive networks, i.e. a higher anti-correlation between the DMN, and the FPN and SN, and 2) CT increases efficiency, centrality and clustering of the FPN, SN and DMN. Lastly, we hypothesized that CT-related neural network changes are related to changes in cognitive performance.

## 2 Materials and methods

### 2.1 Participants

This study was part of the randomized controlled clinical trial ‘COGTIPS’ (COGnitive Training In Parkinson Study; ClinicalTrials.gov registration NCT02920632). For a detailed overview of the methodology we refer to the protocol article (van Balkom et al., 2019) and results on the primary clinical outcomes are reported here (van Balkom et al., 2021 (preprint)). The analysis plan of the current fMRI study was preregistered at the Open Science Framework (registration: osf.io/3st82).

From the full sample of COGTIPS, a sub-sample underwent an MRI scan. General inclusion criteria for participation were 1) mildly to moderately advanced idiopathic PD (Hoehn & Yahr stage < 4; Hoehn and Yahr, 1967) diagnosed by a neurologist, 2) significant subjective cognitive complaints (PD-Cognitive Functional Rating Scale score > 3; Kulisevsky et al., 2013), and 3) home access to and proficiency in using a computer or tablet with internet. General exclusion criteria were 1) a Montreal Cognitive Assessment score < 22 (Dalrymple-Alford et al., 2010, Nasreddine et al., 2005), 2) indications of current drug- or alcohol abuse (CAGE AID-interview score > 1; Brown and Rounds, 1995, Ewing, 1984), 3) moderate to severe depressive symptoms (Beck Depression Inventory score > 18; Beck et al., 1961), 4) an impulse control disorder (positive screening by diagnostic criteria), 5) psychotic symptoms except for benign hallucinations (positive screening by the Schedule for Assessment of Positive Symptoms – PD; Voss et al., 2013), or 6) a history of traumatic brain injury. Exclusion criteria for participation in the MRI study were 1) presence of metal in the body (e.g., a neurostimulator), 2) pregnancy, or 3) difficulty with or shortness of breath during 60 min of lying still, and 4) after baseline scan a space occupying lesion and/or significant vascular abnormalities (Fazekas > 1).

All procedures were performed according to the declaration of Helsinki and all participants gave written informed consent to participate. The study was approved by the VU University Medical Center medical ethical committee.

### 2.2 Procedure and randomization

After eligibility screening, participants underwent an extensive baseline assessment that entailed neuropsychological testing (see Appendix A.6), questionnaires and MR imaging. An overview of outcomes for this study is listed below and described previously in detail (van Balkom et al., 2019). After baseline assessment, participants were randomized, stratified according to education level (low/average versus high), in 1:1 fashion to the experimental cognitive training group (CT) or the active control group (AC) using random number sequence generated randomization lists. Participants remained blind to their allocated condition throughout the entire study and outcome assessors were blinded for the full duration of their role as assessor. Participants performed their respective intervention at home, from their personal computer or tablet via internet. After the intervention, approximately nine weeks after baseline assessment, participants performed a second assessment that entailed neuropsychological testing (using parallel tests if available), questionnaires and MR imaging.

### 2.3 Interventions

Both interventions entailed 24 online, home-based sessions with a duration of approximately 45 min. Participants were instructed to perform their respective intervention three times a week; we did not specify training days to enhance feasibility. Participants were able to pause the intervention session if, for example, they were interrupted or in ‘wearing-off’ state, limiting task execution. The CT, adapted from the ‘BrainGymmer’ platform, consisted of thirteen adaptive training games that aimed to improve executive function, working memory, processing speed and attention (www.braingymmer.com, a product by Dezzel Media). The AC consisted of three non-adaptive games without an expected training effect (i.e., solitaire, hangman and trivia questions) and was used to correct for non-specific cognitive activity.

### 2.4 Measurements

In this study, we only considered the neuropsychological assessments that improved after CT in the full study sample. We measured change in the processing speed/executive function domain with 1) the reaction time on a computerized version of the Tower of London (ToL) task; a planning/executive function task where the participant has to count the number of bead moves needed to reach a solution configuration from a start configuration, ranging from 1 to 5 moves (i.e., difficulty loads S1-S5), and 2) the Stroop Color-Word Test (SCWT); an interference control/processing speed task that consists of word-reading (SCWT-I), color-naming (SCWT-II) and color-word interference (SCWT-III) components. Accuracy on the ToL (i.e., percentage correctly answered trials) was additionally assessed as this was the primary outcome of the clinical trial. Importantly, these tasks were not part of the CT and any improvements would therefore not simply be due to a learning effect.

We compared cognitive function of our study population with pre-existing – in part Dutch – healthy norm group data (Campo and Morales, 2003, Kessels et al., 2016, Nicholas et al., 1989, Schmand et al., 2012) and classified cognitive function as either cognitively normal (PD-NC), cognitive deficits associated with PD-MCI according to level II Movement Disorder Society (MDS) criteria (Litvan et al., 2012), or cognitive deficits associated with probable PD-D (Emre et al., 2007). We additionally assessed motor symptoms (Unified PD Rating Scale – III; Fahn et al., 1987), disease stage (Hoehn and Yahr), and medication use (levodopa equivalent daily dose; Olde Dubbelink et al., 2013). Subjective cognitive complaints were assessed with the PD-Cognitive Functional Rating Scale and we used additional questionnaires to assess psychiatric symptoms and participants’ expectation of the intervention outcome.

### 2.5 Image acquisition

At both time-points, we performed MRI on a GE Signa HDxT 3 T MRI scanner (General Electric, Milwaukee, U.S.). We equipped the 32-channel head coil with foam pads to maximally immobilize the head and thereby reduce head motion. Resting-state functional MRI (rs-fMRI) was acquired using a 10-minute gradient echo‐planar imaging (EPI) sequence (TR = 2200 ms; TE = 28 ms; 64 × 64 matrix; field of view = 21.1 cm; flip angle = 80°) with 40 ascending slices per volume (3.3 × 3.3 mm in‐plane resolution; slice thickness = 3.0 mm; interslice gap = 0.3 mm), which provided whole‐brain coverage. Participants were instructed to keep their eyes closed, not think about anything in particular, and not fall asleep. Anatomical MRI was acquired using a 3D T1-weighted structural magnetization-prepared rapid acquisition gradient-echo with scan parameters according to the ADNI-3 protocol (TR = 6.9 ms, TI = 900 ms, TE = 3.0 ms, matrix size 256 × 256, 1 mm^3^ isotropic voxels, 168 sections) (Weiner et al., 2017).

### 2.6 Image preprocessing

We corrected for susceptibility induced distortions in the functional image by acquiring scans with a reversed phase-encoding direction and applying topup (Andersson et al., 2003) from the FMRIB Software Library (FSL) software suite (Smith et al., 2004). Anatomical and functional images were subsequently preprocessed using fmriprep (v1.4.0; see Appendix A.1 for the full boilerplate; Esteban et al., 2019). Briefly, an average robust template was created from the T1-weighted structural images at either time point using FreeSurfer 6.0.1 (Reuter et al., 2012). Using this robust template, brain surfaces were reconstructed and parcellated according to an atlas (see below). We visually inspected the brain surfaces for any defects. Rs-fMRI images from both time points were skull-stripped, realigned and slice-time corrected, and co-registered to the robust template. Noise regressors were extracted per subject for further denoising of the preprocessed functional time-series. Noise-regressors included global signals within the ventricles (CSF) and white matter (WM) and automatically identified motion-related components based on their high-frequency content and correlation with motion parameters using automatic removal of motion artifacts using independent component analysis (ICA-AROMA; Pruim et al., 2015). We removed the first three non-steady state volumes from the fMRI and spatially smoothed the remaining images with a 6 mm full-width half-maximum isotropic, Gaussian kernel. Simultaneous nuisance regression and temporal filtering (0.009 – 0.13 Hz) was performed using Denoiser (github.com/arielletambini/denoiser). Following the benchmark test from Parkes and colleagues (Parkes et al., 2018) we regressed out all motion-related components identified by ICA-AROMA and eight tissue-averaged physiological regressors: averaged signal in the WM and CSF, along with their temporal derivatives, squares and derivatives squared. No global signal regression was applied. We assessed framewise displacement (FD), computed with fmriprep, as a measure for motion and excluded patients with a liberal motion cut-off of FD_mean_ > 0.5 mm. We additionally assessed differences in image quality metrics calculated with MRIqc (DVARS, entropy-focus criterion, full-width half maximum smoothness and temporal signal-to-noise ratio; Esteban et al., 2017) across groups and time-points (see Appendix A.2).

### 2.7 Timeseries extraction

To extract brain region specific timeseries, we parcellated cortical brain areas into 300 regions according to the Schaefer atlas, which has specifically been developed to match a widely-used seven-network human resting-state network parcellation (Schaefer et al., 2018). The cortical brain areas were derived from registering the Schaefer atlas to FreeSurfer space and we added 14 subcortical areas segmented using FreeSurfer (Fischl et al., 2002) leading to a total of 314 brain areas. This parcellation was registered to the preprocessed and denoised functional images in T1-weighted space using AFNI’s 3Dresample. Quality of the registration was visually inspected. Because EPI distortions around air-tissue boundaries can lead to signal drop-out, we applied a mask to the functional image (Meijer et al., 2017) and excluded brain regions with < 4 active voxels in any participant, prior to timeseries extraction (Vriend et al., 2020). Fourteen brain areas were excluded due to signal drop-out leading to a total of 300 brain regions common across subjects and time points (Appendix A.3).

### 2.8 Resting-state fMRI outcome measures

#### 2.8.1 Between-network connectivity measures

To assess connectivity between the FPN, DMN, and SN, we computed the average Fisher *r*-to-*z* transformed Pearson correlation of the resting-state fMRI time-series between the nodes that belonged to these resting-state networks, based on the network parcellation by Yeo and colleagues (2011). We designated the ventral attention network from the Yeo et al parcellation as SN. We used Pearson correlations to replicate earlier functional connectivity studies (as opposed to wavelet coherence, see below). We did not use the absolute value of the correlations, to maintain valuable information about potential between-network anti-correlations. As indicated in the preregistration, we assessed connectivity between other resting-state networks, i.e., the dorsal attention, limbic, motor and visual networks as exploratory outcomes but do not present these findings here due to space limitations.

#### 2.8.2 Graph measures

Weighted, fully connected (i.e., non-thresholded) connectivity matrices for the calculation of graph indices were computed using a wavelet coherence method in the frequency range *f* = [0.009, 0.08] (Grinsted et al., 2004). This method and frequency range are less contaminated by head motion compared to Pearson correlation connectivity matrices, while the test–retest reliability is better compared with partial correlation coefficient connectivity matrices (Mahadevan et al., 2020).

We assessed global network integration, segregation and connectivity between modules using the following respective measures: a) global efficiency (GE): the inverse of the mean shortest (characteristic) path length in the network (Rubinov and Sporns, 2010), b) modularity (Q): the degree to which a network can be divided in sub-communities (i.e., modules), using modularity maximization with a generalized Louvain method for community detection (Lucas et al., 2011-2019), c) average participation coefficient (PC): the degree to which a node is connected with other communities than its own. For the FPN, DMN and SN, we additionally computed a) efficiency (E, as described above), b) clustering coefficient (CC): the fraction of a node’s neighbors that are also neighbors of each other, and c) normalized betweenness centrality (BC): the average sub-network fraction of shortest paths in a network that pass through a node, normalized by the size of the network. We performed exploratory analyses on the absolute Fisher *r*-to-*z* transformed Pearson correlation connectivity matrices using the same graph indices to assess reliability of the results and enhance comparability with previous research. We additionally assessed the effects of CT on rich club (Alstott et al., 2014) and diverse club coefficients (Bertolero et al., 2017), see Supplemental material 4 for methodological details.

### 2.9 Analyses

We enrolled 140 participants in the overall clinical trial to detect effects at the behavioral level (van Balkom et al., 2019). Based on earlier studies that showed changes in functional activity and connectivity preceding cognitive decline in PD (de Bondt et al., 2016, Gerrits et al., 2015, Trujillo et al., 2015a), neuroimaging indices seem more sensitive to change compared with (global) cognitive tests – and effect sizes are likely larger. Therefore, we enrolled a subgroup consisting of 86 participants for the MRI sub-study.

We performed analyses on the intention-to-treat population (all correctly enrolled and randomized participants). Group differences in demographic and clinical variables were analyzed with the appropriate tests, i.e., Student’s t-tests, Mann-Whitney U tests or Fisher’s exact tests.

For analysis of the abovementioned neuroimaging indices, we used univariate mixed-model analyses to assess the effect of CT relative to AC. We used the after training outcome (T1) as dependent variable, the group (CT vs. AC) as independent variable and the baseline outcome (T0) as covariate. We additionally performed these analyses correcting for age, years of education and sex. For analysis of global graph measures, we considered a significance level of α = 0.05 significant. To correct for multiple comparisons in sub-network analyses we used a D/AP-Sidak adjustment that takes into account the mutual correlation between outcome measures (computed using https://www.quantitativeskills.com/sisa/calculations/bonhlp.htm; Sankoh et al., 1997). For between-network connectivity analyses, we computed an α corrected for three comparisons, adjusted for the correlation r = 0.544 between the three indices after training (α_BN_ = 0.031). For sub-network graph analyses, we computed a separate α for each graph measure adjusted for the correlation between the respective sub-network measures at baseline (r_FPN_ = 0.387, α_FPN_ = 0.026; r_DMN_ = 0.408, α_DMN_ = 0.026; r_SN_ = 0.413, α_SN_ = 0.027). We replicated the frequentist analyses using a Bayesian approach to quantify strength of evidence for either hypothesis (H_0_ or H_1_) using linear models with Monte Carlo integration (500,000 iterations) from the BayesFactor R package. We considered a Bayes Factor B > 3 as substantial evidence for H_1_ and conversely B < 1/3 as substantial evidence for H_0_ (Dienes, 2014). We additionally performed sub-group analyses based on the cognitive status of participants, i.e., PD-NC, PD-MCI or PD-D, by adding an additional covariate with relevant contrasts to the analyses described above. Lastly, we performed post-hoc analyses to assess functional connectivity and graph properties of key regions of the studied brain networks. The evaluated key regions were based on previous literature (Menon, 2011, Seeley et al., 2007, Uddin et al., 2009). Only those regions that showed a significant correlation across groups with neuropsychological outcomes at baseline were considered in the analyses on the effects of cognitive training (see Appendix A.11). We additionally examined connectivity of these nodes with subcortical areas, specifically the caudate nucleus, thalamus and hippocampus, as impaired cortico-subcortical connections are implicated in PD and these subcortical areas are particularly implicated in cognitive function (see Appendix A.11; Ekman et al., 2012, Kandiah et al., 2014, Owen, 2004).

Multivariate (ToL) and univariate (SCWT) linear mixed-model analyses were used to assess differences between groups on the neuropsychological outcomes, as discussed in detail elsewhere (van Balkom et al., 2019). Participants were excluded from ToL data analysis if they showed poor understanding of the task, operationalized as load 1 score < 75%.

The association between change in resting-state functional connectivity and change in cognitive function was analyzed with repeated-measures correlation analyses using the R package ‘rmcorr’ (Bakdash and Marusich, 2017). We assessed the repeated-measures correlation between the neuroimaging outcomes (15 in total) and cognitive outcomes. We analyzed the average ToL reaction time over S1-S5 and the SCWT card I-III separately. Associations with ToL accuracy change were analyzed in an exploratory fashion as this was the primary outcome of the trial. We used an α corrected for multiple comparisons per cognitive test, adjusted for the baseline association between the neuroimaging indices analyzed (similar to described above).

## 3 Results

### 3.1 Participants

**Fig. 1:**
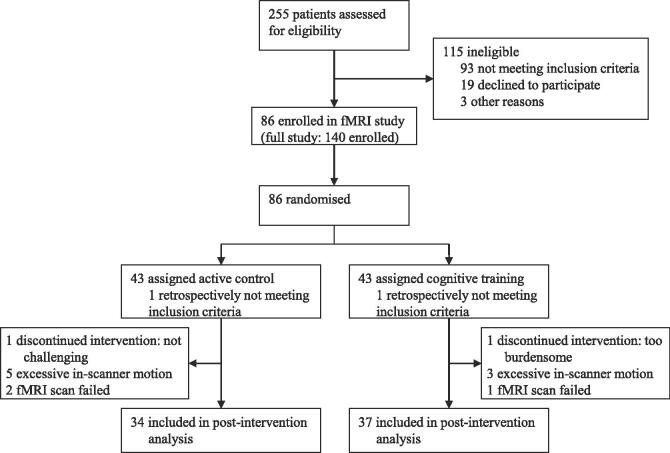
Flowchart of participants.

In COGTIPS, we enrolled and randomized 86 participants in the fMRI study (out of the full sample of 140 participants). The fMRI sub-sample was representative of the full sample concerning demographic characteristics but had a shorter disease duration; for a comparison see Appendix A.5. The participants were evenly distributed across both conditions. A total of thirteen participants (AC n = 8, CT n = 5) were excluded from the analyses due to discontinuation of the intervention, excessive in-scanner motion or fMRI scan failure (see Fig. 1). Of the remaining participants, the groups were evenly matched on demographic and clinical characteristics except for baseline subjective cognitive complaints (see Table 1). Both groups performed similarly on neuropsychological tests. For the entire sample, the cognitive performance on attention and processing speed tasks was, on average, below norm scores, but participants still scored within normal range for other cognitive domains (see Appendix A.6). Inspection of fMRI data quality showed no group differences in motion, but did show, independent of time, a higher temporal derivative of root mean square variance over voxels (DVARS) and entropy-focus criterion (EFC), indicating better average image quality, in the CT group (Appendix A.2).

**Table 1:** Demographic and clinical characteristics of the intention-to-treat population.

	**Active control (n = 34)**	**Cognitive training (n = 37)**	**Group comparison**
**Sex (N (%))**			*p* = .227^‡^
**Male**	23 (68%)	19 (51%)	
**Female**	11 (32%)	18 (49%)	
**Age (years)**	63.8 (6.1)	63.2 (8.3)	t = 0.345, *p* = .731
**Education (years)**	16.7 (4.3)	15.5 (3.6)	t = 1.257, *p* = .213
**Education classification (N (%))^†^**			U = 575, *p* = .514
**3**	0 (0%)	1 (2.7%)	
**4**	2 (5.9%)	3 (8.1%)	
**5**	7 (20.6%)	10 (27.0%)	
**6**	15 (44.1%)	12 (32.4%)	
**7**	10 (29.4%)	11 (29.7%)	
**Disease duration (years, median [range])**	4 [1–16]	4 [0–13]	U = 597, *p* = .710
**UPDRS-III**	19.4 (9.2)	20.6 (9.2)	t = -0.555, *p* = .581
**Hoehn & Yahr stage (N (%))**			U = 581, *p* = .557
**1**	2 (5.9%)	3 (8.1%)	
**1.5**	1 (2.9%)	4 (10.8%)	
**2**	17 (50.0%)	15 (40.5%)	
**2.5**	9 (26.5%)	11 (29.7%)	
**3**	5 (14.7%)	4 (10.8%)	
**LEDD (median [range])**	795 [0–1790]	630 [80–1665]	U = 538.5, *p* = .297
**Medication change during study (N (%))**	8 (7.2%)	7 (7.8%)	*p* = .773^‡^
**LEDD T1 (median [range])**	787 [0–1790]	630 [80–1530]	U = 537, *p* = .289
**MoCA**	26.2 (2.5)	26.6 (1.7)	t = -0.777, *p* = .440
**Global cognitive function classification (N (%))**			*p* = .379^‡^
**Normal cognition**	8 (23.5%)	11 (29.7%)	
**Single-domain MCI**	5 (14.7%)	6 (16.2%)	
**Multi-domain MCI**	13 (38.2%)	17 (45.9%)	
**PD dementia**	8 (23.5%)	3 (8.1%)	
**BDI**	8.4 (3.8)	8.0 (4.3)	t = 0.395, *p* = .694
**QUIP-RS (N = 70)**	20.5 (12.7)	16.1 (13.1)	t = 1.411, *p* = .163
**PAS**	11.6 (7.4)	9.2 (6.2)	t = 1.471, *p* = .146
**AS**	14.2 (4.3)	12.9 (4.3)	t = 1.250, *p* = .216
**Credibility-Expectancy (N = 70)**	31.3 (6.4)	33.1 (6.7)	t = -1.168, *p* = .247
**PD-CFRS (median [range])**	10 [4–22]	7 [3–18]	**U = 422, *p* = .017**
**Compliance (%, median [range])**	100 [71–100]	100 [92–100]	U = 642.5, *p* = .864
**T0-to-T1 interval (days)**	64.7 (7.3)	63.8 (5.0)	t = 0.608, *p* = .545
Data are mean (SD) unless otherwise specified. ^†^According to Verhage education classification.^29 ‡^Fisher’s exact test.*Abbreviations*: AS = Apathy Scale; BDI = Beck Depression Inventory; PAS = Parkinson Anxiety Scale; PD-CFRS = Parkinson’s Disease – Cognitive Functional Rating Scale; LEDD = Levodopa equivalent daily dosage; MCI = mild cognitive impairment; MoCA = Montreal Cognitive Assessment; QUIP-RS = Questionnaire for Impulsive-Compulsive Disorders in Parkinson's Disease – Rating Scale; UPDRS = Unified Parkinson’s Disease Rating Scale.			

Behavioral training effects in the fMRI sample were different compared with the effects in the full study sample. There were no significant positive training effects on the ToL reaction time and SCWT in the current subsample, in contrast with the full study sample. In the current subsample there were trend-significant to significant group differences on the ToL accuracy in favor of the AC, while these were not present in the full study sample (see Appendix A.7).

### 3.2 Preregistered analyses

#### 3.2.1 Main outcomes: Functional connectivity and network topology differences

**Fig. 2:**
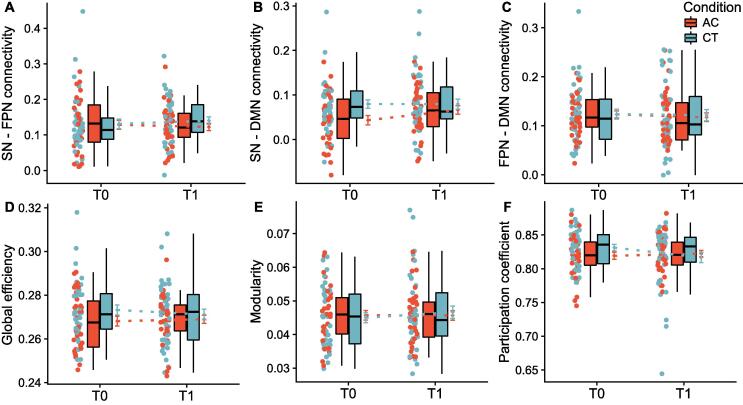
No between-group differences on between-network connectivity (panels A-C) and global network topology (panels D-F).

Univariate mixed-model analyses of connectivity between the SN, FPN and DMN did not reveal significant group differences after training (Fig. 2a-c). Analysis of global efficiency, modularity and participation coefficient at the global network level showed no group differences after training (Fig. 2d-f). Efficiency, clustering coefficient and betweenness centrality at sub-network level did also not differ between groups after training. Bayes factors indicated effects generally in favor of the null hypothesis. All statistics are reported in Table 2. For replicability we performed the same analyses using graph outcomes calculated with Pearson correlation-based connectivity matrices; these analyses resulted in similar findings and results are provided in Appendix A.8.

**Table 2:** Group differences, corrected for baseline value, on primary neuroimaging outcomes.

		**Between-network connectivity**							
		**Crude models**				**Adjusted models**^†^			
		B [SE]	95% CI	p-value	Bayes Factor	B [SE]	95% CI	p-value	Bayes Factor
**SN – FPN**		0.017 [0.014]	−0.011 to 0.046	0.227	0.453 ± 0.12%	0.019 [0.014]	−0.010 to 0.048	0.197	0.494 ± 0.25%
**SN – DMN**		0.004 [0.015]	−0.025 to 0.033	0.791	0.267 ± 0.12%	0.006 [0.014]	−0.023 to 0.034	0.695	0.284 ± 0.21%
**FPN – DMN**		0.004 [0.014]	−0.024 to 0.032	0.771	0.254 ± 0.13%	0.000 [0.014]	−0.028 to 0.029	0.981	0.251 ± 0.27%
		**Graph outcomes**							
		**Crude models**				**Adjusted models**^†^			
		B [SE]	95% CI	p-value	Bayes Factor	B [SE]	95% CI	p-value	Bayes Factor
**Global**	**GE**	0.002 [0.003]	−0.005 to 0.008	0.624	0.277 ± 0.14%	0.002 [0.003]	−0.005 to 0.008	0.622	0.286 ± 0.3%
	**Q**	0.001 [0.002]	−0.003 to 0.006	0.568	0.279 ± 0.12%	0.000 [0.002]	−0.004 to 0.004	0.911	0.253 ± 0.19%
	**PC**	−0.010 [0.009]	−0.028 to 0.007	0.249	0.410 ± 0.11%	−0.007 [0.009]	−0.025 to 0.011	0.435	0.322 ± 0.22%
**FPN**	**GE**	−0.003 [0.007]	−0.017 to 0.012	0.710	0.264 ± 0.14%	−0.003 [0.007]	−0.018 to 0.012	0.682	0.263 ± 0.3%
	**CC**^‡^	0.124 [3.364]	−6.584 to 6.832	0.971	0.246 ± 0.14%	0.973 [3.396]	−5.798 to 7.744	0.775	0.256 ± 0.3%
	**BC**^‡^	0.050 [0.078]	−0.106 to 0.205	0.526	0.287 ± 0.12%	0.062 [0.078]	−0.094 to 0.217	0.430	0.308 ± 0.24%
**DMN**	**GE**	−0.002 [0.007]	−0.016 to 0.012	0.759	0.245 ± 0.14%	−0.003 [0.007]	−0.017 to 0.010	0.638	0.253 ± 0.25%
	**CC**^‡^	0.444 [3.342]	−6.221 to 7.108	0.895	0.252 ± 0.14%	0.651 [3.404]	−6.137 to 7.439	0.849	0.259 ± 0.33%
	**BC**^‡^	0.005 [0.059]	−0.112 to 0.123	0.931	0.245 ± 0.12%	−0.016 [0.056]	−0.128 to 0.097	0.778	0.256 ± 0.19%
**SN**	**GE**	−0.006 [0.011]	−0.027 to 0.016	0.606	0.729 ± 0.13%	−0.010 [0.011]	−0.031 to 0.012	0.381	0.563 ± 0.28%
	**CC**^‡^	−1.593 [3.535]	−8.640 to 5.455	0.654	0.266 ± 0.13%	−1.969 [3.571]	−9.090 to 5.152	0.583	0.272 ± 0.28%
	**BC^‡^**	−0.001 [0.095]	−0.190 to 0.188	0.994	0.245 ± 0.14%	−0.036 [0.095]	−0.226 to 0.154	0.707	0.260 ± 0.28%
^†^Corrected for age, sex, education in years and, for between-network connectivity analyses, framewise displacement. ^‡^Statistics multiplied by 10^3^ because of small values. Abbreviations: BC – Betweenness centrality; CC – Clustering coefficient; DMN – Default mode network; FPN – Frontoparietal network; GE – Global efficiency; PC – Participation coefficient; Q – Modularity; SN – Salience network.									

#### 3.2.2 Exploratory outcomes

To assess the association between functional connectivity or network topology change and change on the ToL or SCWT, we performed group-wise repeated-measures correlation analyses. In both groups, no significant associations were present between change on ToL accuracy or reaction time, or SCWT performance, and functional connectivity or network topology when correcting for multiple comparisons (α = 0.007; see Appendix A.9).

We performed sub-group training effect analyses, distinguishing effects between patients with PD-NC (n = 19), PD-MCI (n = 41) or PD-D (n = 11). Only in the PD-NC sub-sample there was a post-intervention difference between groups, showing higher modularity (B[SE]: 0.008 [0.004], p = .033) and lower participation coefficient (B[SE]: -0.042 [0.015], p = .007) in the CT group, suggesting a more segregated network topology. These effects were, however, not associated with change in neuropsychological test performance. At sub-network level, an isolated between-group difference in the PD-D sub-sample was found showing higher post-training betweenness centrality in the CT versus the AC group, that did not survive correction for multiple comparisons. Note that the subsample sizes were small, particularly for the PD-D subgroup, which limits the reliability of these results. No between-network functional connectivity differences were present (see Appendix A.10).

There were no group differences in the normalized clubness coefficients after intervention, adjusted for baseline values – rich club coefficient: B[SE]: -0.006 [0.019], p = .767, diverse club coefficient: B[SE]: -0.030 [0.031], p = .336.

### 3.3 Post-hoc analyses (not preregistered)

**Fig. 3:**
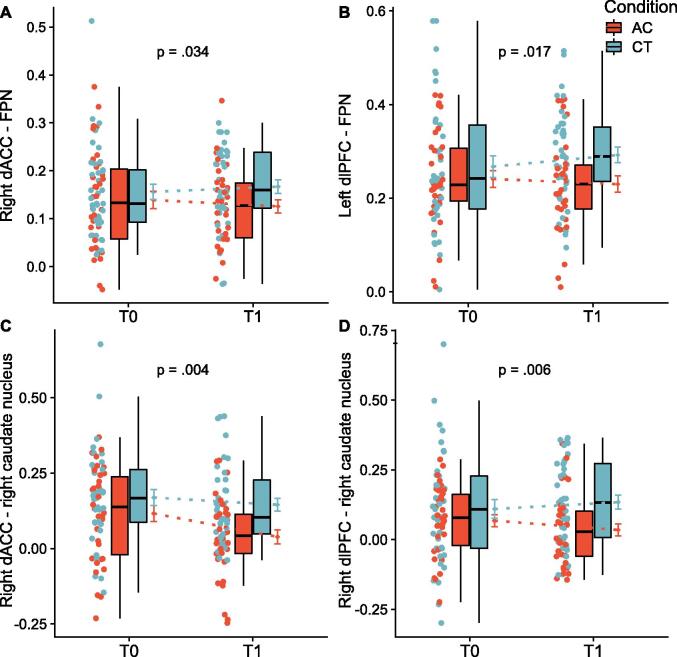
Group differences in connectivity between the dorsal anterior cingulate cortex (dACC) and frontoparietal network (FPN, panel A) and right caudate nucleus (panel C) and the dorsolateral prefrontal cortex (dlPFC) and the FPN (panel B) and right caudate nucleus (panel D). P-values mark difference at post-training corrected for baseline value.

Post-hoc, we performed nodal analyses on the functional connectivity and topology of sub-network key regions (see Appendix A.11), as averaging across network nodes could potentially obscure more localized effects. We found group differences indicative of increased connectivity in the CT group between the right dorsal anterior cingulate cortex (dACC) and the FPN, and of the left dorsolateral prefrontal cortex (dlPFC) within the FPN, that did, however, not survive correction for multiple comparisons (Fig. 3a-b). Because the dACC and dlPFC are implicated in associative cortico-striato-thalamo-cortical circuits we further inspected connectivity of these nodes with subcortical areas. Results suggested group connectivity differences of both nodes predominantly with the right caudate nucleus, indicative of stable/increased connectivity in the CT group versus decreased/stable connectivity in the AC group (see Fig. 3c-d). Analysis of key region nodal participation coefficient, clustering coefficient and betweenness centrality showed no group differences after training.

## 4 Discussion

There is an increasing need for treatment options for cognitive impairment in PD as a large majority of individuals with PD ultimately develops dementia. CT has been proposed as a non-pharmacological treatment option and earlier *meta*-analyses have suggested positive effects on executive functions, but the neural correlates remain largely unknown. In this study, we assessed the effect of eight-week online multi-domain CT on the functional brain network by studying large-scale brain networks in the largest sample of PD patients to date. Our results do not support the hypothesis that CT enhances the functional neural system on a global level; rather, Bayes factors showed effects in favor of no group difference on global outcomes (i.e., null hypothesis). Post-hoc analyses, however, suggested that CT may improve specific functional connections, notably in cortico-cortical and cortico-subcortical circuits that are prominently implicated in PD.

In this study, we used functional connectivity and graph theoretical measures to map functional brain network alterations after CT. While earlier studies in multiple sclerosis and Alzheimer’s disease that applied a multi-domain CT of similar length (4–12 weeks) showed that functional connectivity within networks increased (Bonavita et al., 2015, De Marco et al., 2018, Filippi et al., 2012), our intervention did not affect global and sub-network brain topology. Two of these studies also used active comparators, albeit potentially less engaging control conditions (i.e., news-paper reading and social interaction) compared with ours. One study in individuals with amnestic mild cognitive impairment that studied a 26-week multi-domain CT relative to a documentary-watching control condition showed enhanced segregation between task-positive and task-negative networks (Suo et al., 2016). In contrast to earlier studies, we used an empirically-based, widely-used network parcellation (Yeo et al., 2011), instead of independent component analysis to overcome potential replicability limitations of data-driven methods.

The subtle positive CT effects on executive speed of processing in our full study sample did not reach significance in this sub-sample, potentially due to the smaller size of the fMRI sub-sample as effect estimates were comparable. The lack of robust behavioral CT effects may in the first place explain the absence of brain network alterations, especially at the large-scale network and connectome level. On the other hand, our regional post-hoc findings may reflect a subtle, but still relevant CT effect. That is, functional MRI may be more sensitive in measuring subtle cognitive change than traditional neuropsychological tasks, based on our previous studies that identified functional activity and connectivity changes that preceded decline on neuropsychological test performance in *de novo* PD patients (Gerrits et al., 2015, Trujillo et al., 2015a). Importantly, these results additionally fit the localized CT-induced changes in structural connectivity in this COGTIPS sample, that was additionally correlated with CT-related acceleration of mental processing (Vriend et al., 2021).

To our knowledge, this is the first study on the effect of CT on brain network topology in PD patients. Although there is ample evidence for the existence of network topological alterations already early in PD, therapeutic effects on these alterations are still relatively unexplored in PD – as it is in CT literature. Earlier studies – although mainly in healthy young subjects – have confirmed that CT is able to alter network topology (Finc et al., 2020, Han et al., 2020, Langer et al., 2013, Roman et al., 2017), and modularity may be an important predictor of therapeutic success (Arnemann et al., 2015, Baniqued et al., 2017, Gallen et al., 2016). When we added contrasts to assess CT effects per cognitive status, cognitive training did seem to decrease participation coefficient while modularity increased in cognitively normal individuals – although the latter result did not survive correction for multiple comparisons. These effects, indicating a more segregated network, are associated with more healthy brain topology (Bertolero et al., 2018). The subgroup was very small, however. These results should therefore be considered exploratory to possibly direct future studies to focus on a (larger) subgroup of exclusively PD-NC, PD-MCI or PD-D patients.

To ensure that potential effects were not levelled out by our method of averaging connectivity strength across network nodes, we analyzed connectivity of specific network nodes that previously have been reported to play a key role in the respective networks. The results of these analyses did replicate the findings from earlier studies and revealed localized changes in cortico-cortical and cortico-subcortical connections of the dorsal ACC and dlPFC, key regions of the SN and FPN, respectively. First, it should be noted that these analyses were performed post-hoc and replication of these findings is needed. Our results support multiple earlier findings in neurodegenerative diseases and healthy aging that showed CT-induced connectivity alterations of the ACC (Parisi et al., 2014, Ross et al., 2018, Suo et al., 2016) and dlPFC (Cao et al., 2016, Díez-Cirarda et al., 2016, Ross et al., 2018) using seed-based analysis methods. These structures are critically involved in cognition, especially executive function and this pattern of results thus fits the focus of our experimental intervention on executive functions, mental speed and attention.

In the dACC and dlPFC, CT induced increased average connectivity with FPN nodes. These connectivity changes may indicate that executive function and cognitive control networks alter on a more localized level: first, the dACC is a large structure with monitoring, controlling and evaluating functions and its connections to frontal and parietal areas are important for cognition and – specifically – executive control (Harding et al., 2015, Heilbronner and Hayden, 2016). Second, increased connectivity of the left dlPFC with FPN nodes may exhibit enhanced intrinsic connectivity of this control network that is primarily associated with cognitive control and working memory and exerts top-down control of attention (Dosenbach et al., 2007, Harding et al., 2015). After CT, functional connectivity of the right dlPFC and dorsal ACC with the ipsilateral caudate nucleus was additionally higher compared with the AC group. These cortico-subcortical connections belong to the highly dopamine-dependent cortico-striato-thalamo-cortical circuits involved in executive function (Groenewegen and Uylings, 2010, Owen, 2004) and cognitive control (Peters et al., 2016). Our data showed that functional connectivity with the caudate nucleus was higher post-training in the CT group, with stable or increased connectivity in the CT group relative to a decrease in the AC group connectivity. These post-hoc findings were similar to our diffusion weighted imaging analyses that showed no CT-induced changes in the structural connectome, but did suggest changes in the white matter microstructure in the anterior limb of the internal capsule connecting subcortical structures (Vriend et al., 2021). Taken together, these findings cautiously suggest that CT may influence local rather than global connectivity, specifically those connections that are generally impaired in PD.

Despite the fact that the cortico-cortical and cortico-subcortical connectivity changes and the network topological changes in de PD-NC group fit the existing literature, these results need to be taken with caution as these were post-hoc analyses with no significant association with training-induced change in cognitive performance. The clinical relevance of these changes remains therefore speculative. Additionally, although our earlier systematic review of neural correlates of CT showed that it induced diffuse functional connectivity alterations across the cortex rather than targeted at specific brain areas (van Balkom et al., 2020), the small behavioral effects in our sample retrospectively make the existence of such large-scale effects on brain topology and connectivity inconceivable. It is conceivable, however, that these effects may generalize to large-scale network connectivity and topology if CT is more efficacious and its durability enhanced, e.g., with extended training duration, addition of booster sessions or adjuvant neurostimulation such as repetitive transcranial magnetic stimulation.

This is the largest study yet on the neuroimaging effects of CT in PD. The comprehensive set of clinical, neuropsychological and imaging data provide a complete picture of the potential changes induced by CT. Additionally, in our study we used – as far as available – best practice methods to map neuropsychological test performance (Litvan et al., 2012), preprocessing pipelines (Esteban et al., 2019), definition of the connectivity matrix (Mahadevan et al., 2020), and resting-state network parcellation (Yeo et al., 2011). We additionally used a wavelet coherence method to compute connectivity matrices, with the advantage of being less susceptible to in-scanner head movement, but most earlier studies applying graph theory used Pearson correlational methods which may induce (small) differences in results. Our proof-of-concept analyses using graph outcomes calculated with Pearson correlation connectivity matrices did, however, not provide substantively different results.

A limitation of our study was the prevalence of a small number of participants with possible PD-D on the basis of the full diagnostic criteria (Emre et al., 2007), despite our aim to exclude individuals with severe cognitive impairment by using previously reported optimal diagnostic screening criteria for PD-D on the basis of Montreal Cognitive Assessment score (Dalrymple-Alford et al., 2010). Second, we a-priori assumed the effects of the AC to be non-specific, but this intervention might have induced behavioral or neuroplastic effects that we were not able to identify without a waiting-list control group. Third, the groups were slightly unbalanced on subjective cognitive complaints. Image quality was higher in the CT group which may have affected the validity of the AC group fMRI indices, but it is important to note that these image quality measures are calculated before preprocessing, and nuisance regression and ICA-AROMA has been shown to be a robust method to reduce motion-related artefacts. Lastly, our participants moved considerably during the fMRI scan; we excluded the participants with extreme movement, but for pragmatic reasons our cut-off was quite liberal (Parkes et al., 2018).

In conclusion, we studied the effect of eight-week online multi-domain CT on functional connectivity and brain network topology in individuals with PD. We did not find evidence for alterations in connectivity between large-scale networks or brain network topology. Analogous to effects on cognitive performance, CT effects on network function were small, at the most targeting regional connectivity in fronto-striatal circuits. Post-hoc results hinted at increased segregation of global network topology specifically in cognitively intact PD patients, but replication in larger, homogeneous samples is needed.

## Declaration of Competing Interest

The authors declare that they have no known competing financial interests or personal relationships that could have appeared to influence the work reported in this paper.

## References

[b0005] Aarsland D., Creese B., Politis M., Chaudhuri K.R., ffytche D.H., Weintraub D., Ballard C. (2017). Cognitive decline in Parkinson disease. Nature Reviews. Neurology.

[b0010] Alstott J., Panzarasa P., Rubinov M., Bullmore E.T., Vertes P.E. (2014). A unifying framework for measuring weighted rich clubs. Sci. Rep..

[b0015] Andersson J.L.R., Skare S., Ashburner J. (2003). How to correct susceptibility distortions in spin-echo echo-planar images: application to diffusion tensor imaging. Neuroimage.

[b0020] Arnemann K.L., Chen A.- J.-W., Novakovic-Agopian T., Gratton C., Nomura E.M., D'Esposito M. (2015). Functional brain network modularity predicts response to cognitive training after brain injury. Neurology.

[b0025] Baggio H.-C., Sala-Llonch R., Segura B., Marti M.-J., Valldeoriola F., Compta Y., Tolosa E., Junqué C. (2014). Functional brain networks and cognitive deficits in Parkinson's disease. Hum. Brain Mapp..

[b0030] Baggio H.-C., Segura B., Sala-Llonch R., Marti M.-J., Valldeoriola F., Compta Y., Tolosa E., Junqué C. (2015). Cognitive impairment and resting-state network connectivity in Parkinson's disease. Hum. Brain Mapp..

[b0035] Bakdash J.Z., Marusich L.R. (2017). Repeated Measures Correlation. Front. Psychol..

[b0040] Baniqued P.L., Gallen C.L., Voss M.W., Burzynska A.Z., Wong C.N., Cooke G.E., Duffy K., Fanning J., Ehlers D.K., Salerno E.A., Aguinaga S., McAuley E., Kramer A.F., D'Esposito M. (2017). Brain Network Modularity Predicts Exercise-Related Executive Function Gains in Older Adults. Front. Aging Neurosci..

[b0045] Beck A.T., Ward C.H., Mendelson M., Mock J., Erbaugh J. (1961). An inventory for measuring depression. Arch. Gen. Psychiatry.

[b0050] Bertolero M.A., Yeo B.T.T., Bassett D.S., D’Esposito M. (2018). A mechanistic model of connector hubs, modularity and cognition. Nat. Hum. Behav..

[b0055] Bertolero M.A., Yeo B.T.T., D'Esposito M. (2017). The diverse club. Nature. Communications.

[b0060] Bonavita S., Sacco R., Della Corte M., Esposito S., Sparaco M., d’Ambrosio A., Docimo R., Bisecco A., Lavorgna L., Corbo D., Cirillo S., Gallo A., Esposito F., Tedeschi G. (2015). Computer-aided cognitive rehabilitation improves cognitive performances and induces brain functional connectivity changes in relapsing remitting multiple sclerosis patients: an exploratory study. J. Neurol..

[b0065] Brown R.L., Rounds L.A. (1995). Conjoint screening questionnaires for alcohol and other drug abuse: criterion validity in a primary care practice. Wis. Med. J..

[b0070] Campo P., Morales M. (2003). Reliability and normative data for the Benton Visual Form Discrimination Test. Clin. Neuropsychol..

[b0075] Cao W., Cao X., Hou C., Li T., Cheng Y., Jiang L., Luo C., Li C., Yao D. (2016). Effects of Cognitive Training on Resting-State Functional Connectivity of Default Mode, Salience, and Central Executive Networks. Front. Aging Neurosci..

[b0080] Cerasa A., Gioia M.C., Salsone M., Donzuso G., Chiriaco C., Realmuto S., Nicoletti A., Bellavia G., Banco A., D’amelio M., Zappia M., Quattrone A. (2014). Neurofunctional correlates of attention rehabilitation in Parkinson's disease: an explorative study. Neurol. Sci..

[b0085] Dalrymple-Alford J.C., MacAskill M.R., Nakas C.T., Livingston L., Graham C., Crucian G.P., Melzer T.R., Kirwan J., Keenan R., Wells S., Porter R.J., Watts R., Anderson T.J. (2010). The MoCA: well-suited screen for cognitive impairment in Parkinson disease. Neurology.

[b0090] de Bondt C.C., Gerrits N.J., Veltman D.J., Berendse H.W., van den Heuvel O.A., van der Werf Y.D. (2016). Reduced task-related functional connectivity during a set-shifting task in unmedicated early-stage Parkinson's disease patients. BMC Neuroscience.

[b0095] De Marco M., Meneghello F., Pilosio C., Rigon J., Venneri A. (2018). Up-regulation of DMN Connectivity in Mild Cognitive Impairment Via Network-based Cognitive Training. Curr. Alzheimer Res..

[b0100] Dienes Z. (2014). Using Bayes to get the most out of non-significant results. Front. Psychol..

[b0110] Díez-Cirarda M., Ojeda N., Peña J., Cabrera-Zubizarreta A., Lucas-Jiménez O., Gómez-Esteban J.C., Gómez-Beldarrain M.Á., Ibarretxe-Bilbao N. (2016). Increased brain connectivity and activation after cognitive rehabilitation in Parkinson's disease: a randomized controlled trial. Brain Imag. Behav..

[b0115] Dosenbach N.U.F., Fair D.A., Miezin F.M., Cohen A.L., Wenger K.K., Dosenbach R.A.T., Fox M.D., Snyder A.Z., Vincent J.L., Raichle M.E., Schlaggar B.L., Petersen S.E. (2007). Distinct brain networks for adaptive and stable task control in humans. PNAS.

[b0120] Ekman U., Eriksson J., Forsgren L., Mo S.J., Riklund K., Nyberg L. (2012). Functional brain activity and presynaptic dopamine uptake in patients with Parkinson's disease and mild cognitive impairment: a cross-sectional study. Lancet Neurol..

[b0125] Emre M., Aarsland D., Brown R., Burn D.J., Duyckaerts C., Mizuno Y., Broe G.A., Cummings J., Dickson D.W., Gauthier S., Goldman J., Goetz C., Korczyn A., Lees A., Levy R., Litvan I., McKeith I., Olanow W., Poewe W., Quinn N., Sampaio C., Tolosa E., Dubois B. (2007). Clinical diagnostic criteria for dementia associated with Parkinson's disease. Mov. Disord..

[b0130] Esteban O., Birman D., Schaer M., Koyejo O.O., Poldrack R.A., Gorgolewski K.J., Bernhardt B.C. (2017). MRIQC: Advancing the automatic prediction of image quality in MRI from unseen sites. PLoS ONE.

[b0135] Esteban O., Markiewicz C.J., Blair R.W., Moodie C.A., Isik A.I., Erramuzpe A., Kent J.D., Goncalves M., DuPre E., Snyder M., Oya H., Ghosh S.S., Wright J., Durnez J., Poldrack R.A., Gorgolewski K.J. (2019). fMRIPrep: a robust preprocessing pipeline for functional MRI. Nat. Methods.

[b0140] Ewing J.A. (1984). Detecting alcoholism. The CAGE questionnaire. JAMA.

[b0145] Fahn S., Elton R.L., UPDRS Development Committee, A., Fahn S., Marsden C.D., Calne D.B., Goldstein M. (1987). Recent developments in Parkinson's disease.

[b0150] Filippi M., Riccitelli G., Mattioli F., Capra R., Stampatori C., Pagani E., Valsasina P., Copetti M., Falini A., Comi G., Rocca M.A. (2012). Multiple sclerosis: effects of cognitive rehabilitation on structural and functional MR imaging measures–an explorative study. Radiology.

[b0155] Finc K., Bonna K., He X., Lydon-Staley D.M., Kuhn S., Duch W., Bassett D.S. (2020). Dynamic reconfiguration of functional brain networks during working memory training. Nat. Commun..

[b0160] Fiorenzato E., Strafella A.P., Kim J., Schifano R., Weis L., Antonini A., Biundo R. (2019). Dynamic functional connectivity changes associated with dementia in Parkinson's disease. Brain.

[b0165] Fischl B., Salat D.H., Busa E., Albert M., Dieterich M., Haselgrove C., van der Kouwe A., Killiany R., Kennedy D., Klaveness S., Montillo A., Makris N., Rosen B., Dale A.M. (2002). Whole brain segmentation: automated labeling of neuroanatomical structures in the human brain. Neuron.

[b0170] Galantucci S., Agosta F., Stefanova E., Basaia S., van den Heuvel M.P., Stojković T., Canu E., Stanković I., Spica V., Copetti M., Gagliardi D., Kostić V.S., Filippi M. (2017). Structural Brain Connectome and Cognitive Impairment in Parkinson Disease. Radiology.

[b0175] Gallen C.L., Baniqued P.L., Chapman S.B., Aslan S., Keebler M., Didehbani N., D’Esposito M., Hayasaka S. (2016). Modular Brain Network Organization Predicts Response to Cognitive Training in Older Adults. PLoS ONE.

[b0180] Gerrits N.J.H.M., van der Werf Y.D., Verhoef K.M.W., Veltman D.J., Groenewegen H.J., Berendse H.W., van den Heuvel O.A. (2015). Compensatory fronto-parietal hyperactivation during set-shifting in unmedicated patients with Parkinson's disease. Neuropsychologia.

[b0185] Gorges M., Müller H.-P., Liepelt-Scarfone I., Storch A., Dodel R., Hilker-Roggendorf R., Berg D., Kunz M.S., Kalbe E., Baudrexel S., Kassubek J. (2019). Structural brain signature of cognitive decline in Parkinson's disease: DTI-based evidence from the LANDSCAPE study. Therapeut. Adv. Neurol. Disord..

[b0190] Grinsted A., Moore J.C., Jevrejeva S. (2004). Application of the cross wavelet transform and wavelet coherence to geophysical time series. Nonlinear Processes Geophys..

[b0195] Groenewegen, H., Uylings, H., 2010. Organization of prefrontal-striatal connections. Handbook of basal ganglia structure and function. Academic Press, San Diego.

[b0200] Han K., Chapman S.B., Krawczyk D.C. (2020). Cognitive Training Reorganizes Network Modularity in Traumatic Brain Injury. Neurorehabilit. Neural Repair.

[b0205] Harding I.H., Yucel M., Harrison B.J., Pantelis C., Breakspear M. (2015). Effective connectivity within the frontoparietal control network differentiates cognitive control and working memory. Neuroimage.

[b0210] Heilbronner S.R., Hayden B.Y. (2016). Dorsal Anterior Cingulate Cortex: A Bottom-Up View. Annu. Rev. Neurosci..

[b0215] Hoehn M.M., Yahr M.D. (1967). Parkinsonism: onset, progression and mortality. Neurology.

[b0220] Kandiah N., Zainal N.H., Narasimhalu K., Chander R.J., Ng A., Mak E., Au W.L., Sitoh Y.Y., Nadkarni N., Tan L.C.S. (2014). Hippocampal volume and white matter disease in the prediction of dementia in Parkinson's disease. Parkinson. Related Disord..

[b0225] Kessels R.P.C., Bucks R.S., Willison R.W., Byrne L.M.T. (2016).

[b0230] Kim J., Wilhelm T. (2008). What is a complex graph?. Phys. a-Statist. Mechan. Appl..

[b0235] Kulisevsky J., Fernández de Bobadilla R., Pagonabarraga J., Martínez-Horta S., Campolongo A., García-Sánchez C., Pascual-Sedano B., Ribosa-Nogué R., Villa-Bonomo C. (2013). Measuring functional impact of cognitive impairment: validation of the Parkinson's disease cognitive functional rating scale. Parkinson. Relat. Disord..

[b0240] Laansma, M., Bright, J., Al-Bachari, S., Anderson, T., Ard, T., Assogna, F., Baquero, K., Berendse, H., Blair, J., Cendes, F., Dalrymple-Alford, J., de Bie, R.M.A., Debove, I., Dirkx, M., Druzgal, J., Emsley, H.C.A., Garraux, G., Guimarães, R., Gutman, B., Helmich, R., Klein, J., Mackay, C., McMillan, C., Melzer, T., Parkes, L., Piras, F., Pitcher, T., Poston, K., Rango, M., Ribeiro, L., Rocha, C., Rummel, C., Santos, L.S.R., Schmidt, R., Schwingenschuh, P., Spalletta, G., Squarcina, L., van den Heuvel, O., Vriend, C., Wang, J.-J., Weintraub, D., Wiest, R., Yasuda, C., Jahanshad, N., Thompson, P., van der Werf, Y., 2020. An International Multicenter Analysis of Brain Structure across Clinical Stages of Parkinson's Disease: The ENIGMA-Parkinson's Study. medRxiv. https://doi.org/10.1101/2020.04.28.20072710.

[b0245] Langer N., von Bastian C.C., Wirz H., Oberauer K., Jäncke L. (2013). The effects of working memory training on functional brain network efficiency. Cortex.

[b0250] Leung I.H.K., Walton C.C., Hallock H., Lewis S.J.G., Valenzuela M., Lampit A. (2015). Cognitive training in Parkinson disease: A systematic review and meta-analysis. Neurology.

[b0255] Litvan I., Goldman J.G., Tröster A.I., Schmand B.A., Weintraub D., Petersen R.C., Mollenhauer B., Adler C.H., Marder K., Williams-Gray C.H., Aarsland D., Kulisevsky J., Rodriguez-Oroz M.C., Burn D.J., Barker R.A., Emre M. (2012). Diagnostic criteria for mild cognitive impairment in Parkinson's disease: Movement Disorder Society Task Force guidelines. Mov. Disord..

[b0260] Lucas, G.S.J., Bazzi, M., Inderjit, S.J., Mucha, P.J., 2011-2019. A generalized Louvain method for community detection implemented in MATLAB.

[b0265] Luo C.Y., Guo X.Y., Song W., Chen Q., Cao B., Yang J., Gong Q.Y., Shang H.-F. (2015). Functional connectome assessed using graph theory in drug-naive Parkinson's disease. J. Neurol..

[b0270] Mahadevan, A.S., Tooley, U.A., Bertolero, M.A., Mackey, A.P., Bassett, D.S., 2020. Evaluating the sensitivity of functional connectivity measures to motion artifact in resting-state fMRI data. bioRxiv. https://doi.org/10.1101/2020.05.04.072868. 10.1016/j.neuroimage.2021.118408 34284108

[b0275] Medaglia J.D., Lynall M.E., Bassett D.S. (2015). Cognitive network neuroscience. J. Cognit. Neurosci..

[b0280] Meijer K.A., Eijlers A.J.C., Douw L., Uitdehaag B.M.J., Barkhof F., Geurts J.J.G., Schoonheim M.M. (2017). Increased connectivity of hub networks and cognitive impairment in multiple sclerosis. Neurology.

[b0285] Meng Y.H., Wang P.P., Song Y.X., Wang J.H. (2019). Cholinesterase inhibitors and memantine for Parkinson's disease dementia and Lewy body dementia: A meta-analysis. Exp. Ther. Med..

[b0290] Menon V. (2011). Large-scale brain networks and psychopathology: a unifying triple network model. Trends Cogn Sci.

[b0295] Menon V., Uddin L.Q. (2010). Saliency, switching, attention and control: a network model of insula function. Brain Struct. Funct..

[b0300] Muslimovic D., Post B., Speelman J.D., Schmand B. (2005). Cognitive profile of patients with newly diagnosed Parkinson disease. Neurology.

[b0305] Nasreddine Z.S., Phillips N.A., Bedirian V., Charbonneau S., Whitehead V., Collin I., Cummings J.L., Chertkow H. (2005). The Montreal Cognitive Assessment, MoCA: a brief screening tool for mild cognitive impairment. J. Am. Geriatr. Soc..

[b0310] Nicholas L.E., Brookshire R.H., Maclennan D.L., Schumacher J.G., Porrazzo S.A. (1989). Revised Administration and Scoring Procedures for the Boston Naming Test and Norms for Non-Brain-Damaged Adults. Aphasiology.

[b0315] Nombela C., Bustillo P.J., Castell P.F., Sanchez L., Medina V., Herrero M.T. (2011). Cognitive rehabilitation in Parkinson's disease: evidence from neuroimaging. Front. Neurol..

[b0320] Noufi P., Khoury R., Jeyakumar S., Grossberg G.T. (2019). Use of Cholinesterase Inhibitors in Non-Alzheimer's Dementias. Drugs Aging.

[b0325] Olde Dubbelink K.T., Hillebrand A., Stoffers D., Deijen J.B., Twisk J.W., Stam C.J., Berendse H.W. (2014). Disrupted brain network topology in Parkinson's disease: a longitudinal magnetoencephalography study. Brain.

[b0330] Olde Dubbelink K.T.E., Schoonheim M.M., Deijen J.B., Twisk J.W.R., Barkhof F., Berendse H.W. (2014). Functional connectivity and cognitive decline over 3 years in Parkinson disease. Neurology.

[b0335] Olde Dubbelink K.T.E., Stoffers D., Deijen J.B., Twisk J.W.R., Stam C.J., Berendse H.W. (2013). Cognitive decline in Parkinson's disease is associated with slowing of resting-state brain activity: a longitudinal study. Neurobiol. Aging.

[b0340] Owen A.M. (2004). Cognitive dysfunction in Parkinson's disease: the role of frontostriatal circuitry. The Neuroscientist.

[b0345] Parisi L., Rocca M.A., Valsasina P., Panicari L., Mattioli F., Filippi M. (2014). Cognitive rehabilitation correlates with the functional connectivity of the anterior cingulate cortex in patients with multiple sclerosis. Brain Imag. Behav..

[b0350] Parkes L., Fulcher B., Yucel M., Fornito A. (2018). An evaluation of the efficacy, reliability, and sensitivity of motion correction strategies for resting-state functional MRI. Neuroimage.

[b0355] Pereira J.B., Aarsland D., Ginestet C.E., Lebedev A.V., Wahlund L.-O., Simmons A., Volpe G., Westman E. (2015). Aberrant cerebral network topology and mild cognitive impairment in early Parkinson's disease. Hum. Brain Mapp..

[b0360] Pereira J.B., Svenningsson P., Weintraub D., Bronnick K., Lebedev A., Westman E., Aarsland D. (2014). Initial cognitive decline is associated with cortical thinning in early Parkinson disease. Neurology.

[b0365] Peters S.K., Dunlop K., Downar J. (2016). Cortico-Striatal-Thalamic Loop Circuits of the Salience Network: A Central Pathway in Psychiatric Disease and Treatment. Front. Syst. Neurosci..

[b0370] Pruim R.H.R., Mennes M., van Rooij D., Llera A., Buitelaar J.K., Beckmann C.F. (2015). ICA-AROMA: A robust ICA-based strategy for removing motion artifacts from fMRI data. Neuroimage.

[b0375] Putcha D., Ross R.S., Cronin-Golomb A., Janes A.C., Stern C.E. (2015). Altered intrinsic functional coupling between core neurocognitive networks in Parkinson's disease. Neuroimage Clin.

[b0380] Putcha D., Ross R.S., Cronin-Golomb A., Janes A.C., Stern C.E. (2016). Salience and Default Mode Network Coupling Predicts Cognition in Aging and Parkinson's Disease. J. Int. Neuropsychol. Soc..

[b0385] Reuter M., Schmansky N.J., Rosas H.D., Fischl B. (2012). Within-subject template estimation for unbiased longitudinal image analysis. Neuroimage.

[b0390] Roman F.J., Iturria-Medina Y., Martinez K., Karama S., Burgaleta M., Evans A.C., Jaeggi S.M., Colom R. (2017). Enhanced structural connectivity within a brain sub-network supporting working memory and engagement processes after cognitive training. Neurobiol. Learn. Mem..

[b0395] Ross, L.A., Webb, C.E., Whitaker, C., Hicks, J.M., Schmidt, E.L., Samimy, S., Dennis, N.A., Visscher, K.M., 2018. The Effects of Useful Field of View Training on Brain Activity and Connectivity. J. Gerontol. Series B, Psychol. Sci. Soc. Sci. Doi: 10.1093/geronb/gby041. 10.1093/geronb/gby041 PMC6941209 29757433

[b0400] Rubinov M., Sporns O. (2010). Complex network measures of brain connectivity: uses and interpretations. Neuroimage.

[b0405] Sankoh A.J., Huque M.F., Dubey S.D. (1997). Some comments on frequently used multiple endpoint adjustment methods in clinical trials. Stat. Med..

[b0410] Schaefer A., Kong R., Gordon E.M., Laumann T.O., Zuo X.N., Holmes A.J., Eickhoff S.B., Yeo B.T.T. (2018). Local-Global Parcellation of the Human Cerebral Cortex from Intrinsic Functional Connectivity MRI. Cereb. Cortex.

[b0415] Schmand, B., Houx, P., de Koning, I., 2012. [Norms neuropsychological assessments]. https://www.psynip.nl/en/.

[b0420] Seeley W.W., Menon V., Schatzberg A.F., Keller J., Glover G.H., Kenna H., Reiss A.L., Greicius M.D. (2007). Dissociable intrinsic connectivity networks for salience processing and executive control. J. Neurosci..

[b0425] Seppi K., Weintraub D., Coelho M., Perez-Lloret S., Fox S.H., Katzenschlager R., Hametner E.M., Poewe W., Rascol O., Goetz C.G., Sampaio C. (2011). The Movement Disorder Society Evidence-Based Medicine Review Update: Treatments for the non-motor symptoms of Parkinson's disease. Mov. Disord..

[b0430] Smith S.M., Jenkinson M., Woolrich M.W., Beckmann C.F., Behrens T.E., Johansen-Berg H., Bannister P.R., De Luca M., Drobnjak I., Flitney D.E., Niazy R.K., Saunders J., Vickers J., Zhang Y., De Stefano N., Brady J.M., Matthews P.M. (2004). Advances in functional and structural MR image analysis and implementation as FSL. Neuroimage.

[b0435] Spreng R.N., Mar R.A., Kim A.S. (2009). The common neural basis of autobiographical memory, prospection, navigation, theory of mind, and the default mode: a quantitative meta-analysis. J. Cognit. Neurosci..

[b0440] Stam C.J., Reijneveld J.C. (2007). Graph theoretical analysis of complex networks in the brain. Nonlin. Biomed. Phys..

[b0445] Suo C., Singh M.F., Gates N., Wen W., Sachdev P., Brodaty H., Saigal N., Wilson G.C., Meiklejohn J., Singh N., Baune B.T., Baker M., Foroughi N., Wang Y., Mavros Y., Lampit A., Leung I., Valenzuela M.J. (2016). Therapeutically relevant structural and functional mechanisms triggered by physical and cognitive exercise. Mol. Psychiatry.

[b0450] Svenningsson P., Westman E., Ballard C., Aarsland D. (2012). Cognitive impairment in patients with Parkinson's disease: diagnosis, biomarkers, and treatment. Lancet Neurol..

[b0455] Trujillo J.P., Gerrits N.J.H.M., Veltman D.J., Berendse H.W., van der Werf Y.D., van den Heuvel O.A. (2015). Reduced neural connectivity but increased task-related activity during working memory in de novo Parkinson patients. Hum. Brain Mapp..

[b0460] Trujillo J.P., Gerrits N.J., Vriend C., Berendse H.W., van den Heuvel O.A., van der Werf Y.D. (2015). Impaired planning in Parkinson's disease is reflected by reduced brain activation and connectivity. Hum. Brain Mapp..

[b0465] Uddin L.Q., Clare Kelly A.M., Biswal B.B., Xavier Castellanos F., Milham M.P. (2009). Functional connectivity of default mode network components: correlation, anticorrelation, and causality. Hum. Brain Mapp..

[b0470] van Balkom, T.D., Berendse, H.W., van der Werf, Y.D., Twisk, J.W.R., Peeters, C.F.W., Hagen, R.H., Berk, T., van den Heuvel, O.A., Vriend, C., 2021. Effect of Eight-Week Online Cognitive Training in Parkinson’s Disease: A Randomized Controlled Trial. medRxiv. https://doi.org/10.1101/2021.03.04.21252499. 10.1016/j.parkreldis.2022.02.018 35248830

[b0475] van Balkom T.D., Berendse H.W., van der Werf Y.D., Twisk J.W.R., Zijlstra I., Hagen R.H., Berk T., Vriend C., van den Heuvel O.A. (2019). COGTIPS: a double-blind randomized active controlled trial protocol to study the effect of home-based, online cognitive training on cognition and brain networks in Parkinson's disease. BMC Neurol..

[b0480] van Balkom T.D., van den Heuvel O.A., Berendse H.W., van der Werf Y.D., Vriend C. (2020). The Effects of Cognitive Training on Brain Network Activity and Connectivity in Aging and Neurodegenerative Diseases: a Systematic Review. Neuropsychol. Rev..

[b0485] Voss T., Bahr D., Cummings J., Mills R., Ravina B., Williams H. (2013). Performance of a shortened Scale for Assessment of Positive Symptoms for Parkinson's disease psychosis. Parkinson. Relat. Disord..

[b0490] Vriend C., van Balkom T.D., Berendse H.W., van der Werf Y.D., van den Heuvel O.A. (2021). Cognitive Training in Parkinson's Disease Induces Local, Not Global, Changes in White Matter Microstructure. Neurotherapeutics..

[b0500] Vriend C., van den Heuvel O.A., Berendse H.W., van der Werf Y.D., Douw L. (2018). Global and Subnetwork Changes of the Structural Connectome in de novo Parkinson's Disease. Neuroscience.

[b0505] Vriend C., Wagenmakers M.J., van den Heuvel O.A., van der Werf Y.D. (2020). Resting-state network topology and planning ability in healthy adults. Brain Struct. Funct..

[b0510] Weiner M.W., Veitch D.P., Aisen P.S., Beckett L.A., Cairns N.J., Green R.C., Harvey D., Jack C.R., Jagust W., Morris J.C., Petersen R.C., Salazar J., Saykin A.J., Shaw L.M., Toga A.W., Trojanowski J.Q., Alzheimer's Disease Neuroimaging, I. (2017). The Alzheimer's Disease Neuroimaging Initiative 3: Continued innovation for clinical trial improvement. Alzheimers Dement.

[b0515] Wolters A.F., van de Weijer S.C.F., Leentjens A.F.G., Duits A.A., Jacobs H.I.L., Kuijf M.L. (2019). Resting-state fMRI in Parkinson's disease patients with cognitive impairment: A meta-analysis. Parkinson. Relat. Disord..

[b0520] Yeo B.T., Krienen F.M., Sepulcre J., Sabuncu M.R., Lashkari D., Hollinshead M., Roffman J.L., Smoller J.W., Zöllei L., Polimeni J.R., Fischl B., Liu H., Buckner R.L. (2011). The organization of the human cerebral cortex estimated by intrinsic functional connectivity. J. Neurophysiol..

