# Supplementary material for: Eight-week multi-domain cognitive training does not impact large-scale resting-state brain networks in Parkinson’s disease
Source: Neuroimage Clin. 2022 Jan 30;33:102952. doi: 10.1016/j.nicl.2022.102952 (PMC8819471; doi:10.1016/j.nicl.2022.102952)
Supplement: Supplementary data 1 [file mmc1.pdf]

APPENDIX

Eight-week multi-domain cognitive training does not impact large-scale resting-state brain networks in Parkinson's disease

by

Van Balkom, Van den Heuvel, Berendse, Van der Werf and Vriend

in

NeuroImage: Clinical

Contents

A.1	fmriprep boilerplate	page 2
A.2	Image quality metrics per group and time-point	page 5
A.3	Brain regions used for analyses	page 6
A.4	Rich and diverse club coefficient analysis methods	page 7
A.5	Comparison between fMRI and non-fMRI sample	page 10
A.6	Cognitive performance of the study sample	page 11
A.7	Summary of behavioral results in the fMRI and full study sample	page 12
A.8	Analysis of graph outcomes calculated with Pearson correlation-based connectivity matrices (instead of wavelet coherence-based)	page 14
A.9	Repeated-measures correlation analyses	page 16
A.10	Effects of CT grouped by cognitive status	page 18
A.11	Nodal connectivity and topology of sub-network key regions	page 19
	References	page 22

Appendix A.1 – fMRIPrep boilerplate

Results included in this manuscript come from preprocessing performed using fMRIPrep 1.4.1 (RRID:SCR_016216),[1] which is based on Nipype 1.2.0 (RRID:SCR_002502).[2]

Anatomical data preprocessing

A total of 2 T1-weighted (T1w) images were found within the input BIDS dataset. All of them were corrected for intensity non-uniformity (INU) with N4BiasFieldCorrection,[3] distributed with ANTs 2.2.0 (RRID:SCR_004757).[4] The T1w-reference was then skull-stripped with a Nipype implementation of the antsBrainExtraction.sh workflow (from ANTs), using OASIS30ANTs as target template. Brain tissue segmentation of cerebrospinal fluid (CSF), white-matter (WM) and gray-matter (GM) was performed on the brain-extracted T1w using fast (FSL 5.0.9, RRID:SCR_002823).[5] A T1w-reference map was computed after registration of 2 T1w images (after INU-correction) using mri_robust_template (FreeSurfer 6.0.1).[6] Brain surfaces were reconstructed using recon-all (FreeSurfer 6.0.1, RRID:SCR_001847),[7] and the brain mask estimated previously was refined with a custom variation of the method to reconcile ANTs-derived and FreeSurfer-derived segmentations of the cortical gray-matter of Mindboggle (RRID:SCR_002438).[8] Volume-based spatial normalization to one standard space (MNI152NLin6Asym) was performed through nonlinear registration with antsRegistration (ANTs 2.2.0), using brain-extracted versions of both T1w reference and the T1w template. The following template was selected for spatial normalization: FSL's MNI ICBM 152 non-linear 6th Generation Asymmetric Average Brain Stereotaxic Registration Model (RRID:SCR_002823; TemplateFlow ID: MNI152NLin6Asym).[9]

Functional data preprocessing

For each of the 2 BOLD runs found per subject (across all tasks and sessions), the following preprocessing was performed. First, a reference volume and its skull-stripped version were generated using a custom methodology of fMRIPrep. The BOLD reference was then co-registered to the T1w reference using bbregister (FreeSurfer) which implements boundary-based registration.[10] Co-registration was configured with nine degrees of freedom to account for distortions remaining in the BOLD reference. Head-motion parameters with respect to the BOLD reference (transformation matrices, and six corresponding rotation and translation parameters) are estimated before any spatiotemporal filtering using mcflirt (FSL 5.0.9).[11] BOLD runs were slice-time corrected using 3dTshift from AFNI 20160207 (RRID:SCR_005927).[12] The BOLD time-series, were resampled to surfaces on the following spaces: fsnative, fsaverage5. The BOLD time-series (including slice-timing correction when applied) were resampled onto their original, native space by applying a single, composite transform to correct for head-motion and susceptibility distortions. These resampled BOLD time-series will be

referred to as preprocessed BOLD in original space, or just preprocessed BOLD. The BOLD time-series were resampled into standard space, generating a preprocessed BOLD run in [‘MNI152NLin6Asym’] space. First, a reference volume and its skull-stripped version were generated using a custom methodology of fMRIPrep. Automatic removal of motion artifacts using independent component analysis (ICA-AROMA)[13] was performed on the preprocessed BOLD on MNI space time-series after removal of non-steady state volumes and spatial smoothing with an isotropic, Gaussian kernel of 6mm FWHM (full-width half-maximum). Corresponding “non-aggresively” denoised runs were produced after such smoothing. Additionally, the “aggressive” noise-regressors were collected and placed in the corresponding confounds file. Several confounding time-series were calculated based on the preprocessed BOLD: framewise displacement (FD), DVARS and three region-wise global signals. FD and DVARS are calculated for each functional run, both using their implementations in Nipype (following the definitions by Power et al.).[14] The three global signals are extracted within the CSF, the WM, and the whole-brain masks. Additionally, a set of physiological regressors were extracted to allow for component-based noise correction (CompCor).[15] Principal components are estimated after high-pass filtering the preprocessed BOLD time-series (using a discrete cosine filter with 128s cut-off) for the two CompCor variants: temporal (tCompCor) and anatomical (aCompCor). tCompCor components are then calculated from the top 5% variable voxels within a mask covering the subcortical regions. This subcortical mask is obtained by heavily eroding the brain mask, which ensures it does not include cortical GM regions. For aCompCor, components are calculated within the intersection of the aforementioned mask and the union of CSF and WM masks calculated in T1w space, after their projection to the native space of each functional run (using the inverse BOLD-to-T1w transformation). Components are also calculated separately within the WM and CSF masks. For each CompCor decomposition, the k components with the largest singular values are retained, such that the retained components’ time series are sufficient to explain 50 percent of variance across the nuisance mask (CSF, WM, combined, or temporal). The remaining components are dropped from consideration. The head-motion estimates calculated in the correction step were also placed within the corresponding confounds file. The confound time series derived from head motion estimates and global signals were expanded with the inclusion of temporal derivatives and quadratic terms for each.[16] Frames that exceeded a threshold of 0.5 mm FD or 1.5 standardised DVARS were annotated as motion outliers. All resamplings can be performed with a single interpolation step by composing all the pertinent transformations (i.e. head-motion transform matrices, susceptibility distortion correction when available, and co-registrations to anatomical and output spaces). Gridded (volumetric) resamplings were performed using antsApplyTransforms (ANTs), configured with Lanczos interpolation to minimize the smoothing effects of other kernels.[17] Non-gridded (surface) resamplings were performed using mri_vol2surf (FreeSurfer).

Many internal operations of fMRIPrep use Nilearn 0.5.2 (RRID:SCR_001362),[18] mostly within the functional processing workflow. For more details of the pipeline, see the section corresponding to workflows in fMRIPrep's documentation.

Appendix A.2 – Image quality metrics per group and time-point

Quality of the fMRI data was extracted and compared across groups and time points with the NparLD package in R that allows a non-parametric analysis of longitudinal data.

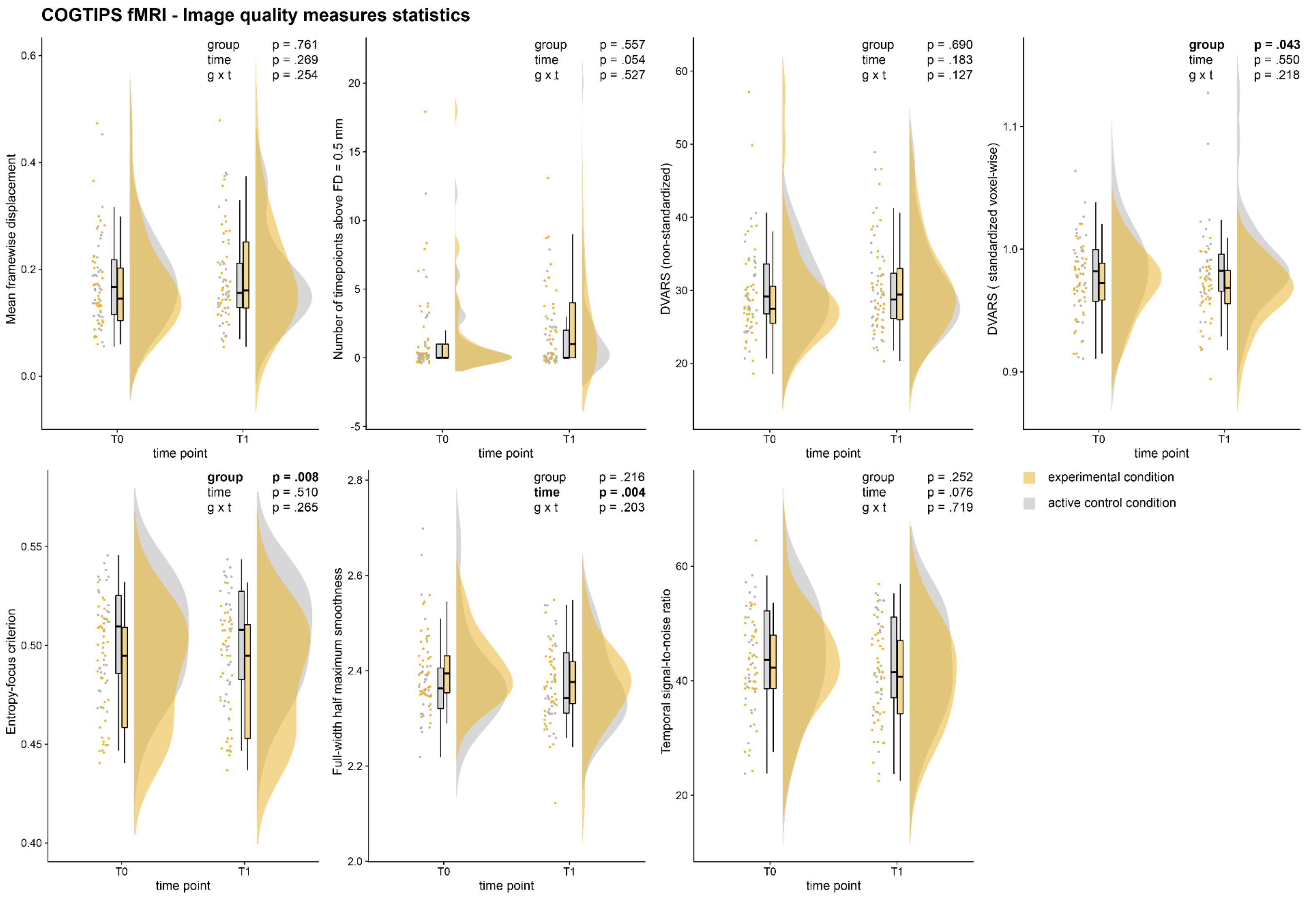

Figure A.1 Group-by-time comparison of image quality metrics. Framewise displacement (FD) was computed with fmriprep; all other metrics with mriqc (see its documentation for a description of the metrics: URL https://mriqc.readthedocs.io/).

Appendix A.3 – Brain regions used for analyses

For functional connectivity and graph analysis we used the brain regions from the Schaefer 7-network and FreeSurfer subcortical atlases, that had adequate quality and were common across all participants and time-points, as listed below.

Table A.1a Overview of nodes included for analyses

Networks of interest			Networks of no interest	
**Salience network nodes**	**Frontoparietal network nodes**	**Default mode network nodes**	**Visual, somatomotor, dorsal attention and limbic network nodes**	**Sub-cortical network nodes (FreeSurfer)**
LH_SalVentAttn_ParOper_1 – 3	LH_Cont_Par_1 – 4	LH_Default_Temp_1, 3 – 8	LH_Vis_1 – 24	Left-Thalamus-Proper
LH_SalVentAttn_TempOcc_1	LH_Cont_Temp_1	LH_Default_Par_1 – 4	RH_Vis_1 – 23	Left-Caudate
LH_SalVentAttn_FrOperIns_1 – 7	LH_Cont_OFC_1	LH_Default_PFC_1 – 18	LH_SomMot_1 – 29	Left-Putamen
LH_SalVentAttn_PFCl_1	LH_Cont_PFCl_1 – 6	LH_Default_pCunPCC_1 – 8	RH_SomMot_1 – 28	Left-Pallidum
LH_SalVentAttn_Med_1 – 4	LH_Cont_pCun_1 – 2	RH_Default_Par_1 – 5	LH_DorsAttn_Post_1 – 12	Left-Hippocampus
RH_SalVentAttn_TempOccPar_1 – 5	LH_Cont_PFCmp_1	RH_Default_Temp_1, 3 – 6	LH_DorsAttn_FEF_1 – 3	Left-Amygdala
RH_SalVentAttn_PrC_1	RH_Cont_Par_1 – 3	RH_Default_PFCv_1 – 3	LH_DorsAttn_PrCv_1	Right-Thalamus-Proper
RH_SalVentAttn_FrOperIns_1 – 6	RH_Cont_Temp_1	RH_Default_PFCdPFCm_1 – 10	RH_DorsAttn_Post_1 – 14	Right-Caudate
RH_SalVentAttn_Med_1 – 6	RH_Cont_PFCv_1	RH_Default_pCunPCC_1 – 6	RH_DorsAttn_FEF_1 – 3	Right-Putamen
	RH_Cont_PFCl_1		RH_DorsAttn_PrCv_1	Right-Pallidum
	RH_Cont_Par_2 – 3		LH_Limbic_TempPole_1 – 7	Right-Hippocampus
	RH_Cont_PFCl_2 – 12		RH_Limbic_OFC_4	Right-Amygdala
	RH_Cont_pCun_1 – 2		RH_Limbic_TempPole_1 – 6	
	RH_Cont_PFCmp_1 – 2			

Table A.1b Overview of nodes excluded for analyses

LH_Cont_Cing_1 – 2	RH_Cont_Cing_1 – 2
LH_Default_Temp_2	RH_Default_Temp_2
LH_Limbic_OFC_1 – 3	RH_Limbic_OFC_1 – 3
Left-Accumbens-area	Right-Accumbens-area

Appendix A.4 – Rich and diverse club coefficient analysis methods

Rich and diverse club coefficient calculation

Rich club and diverse club coefficient were additionally assessed as exploratory outcome. Briefly, the brain's rich club is a set brain regions that is highly and strongly connected within the neural network and is densely interconnected.[19] A rich club organization is associated with cognitive performance - executive function in particular - in healthy elderly.[20] The diverse club, on the other hand, has recently been proposed as a different set of highly connected brain region in the neural network that has diverse connectivity (i.e., high participation coefficient).[21] The diverse club seems is important for integration within the network compared with the rich club and shows increased activity in complex cognitive tasks. We defined the club size by first ranking nodes on strength or participation coefficient and subsequently assessed clubness using a range of percentile cut-offs (highest 30% to highest 5%, see below). Both clubness coefficients (θ_{rich} and $\theta_{diverse}$) were calculated using the following equation for fully weighted networks, as used in previous studies:[21, 22] $\theta = \frac{e}{n\,(n-1)\,/\,2}$, where e is the sum of edge weights between the club's nodes and n is the number of nodes within the club. The normalized clubness coefficients were subsequently calculated by dividing the coefficient by the mean club coefficient of 1000 random networks (as described earlier), indicating that a normalized $\theta > 1$ means larger than random. As for all cut-offs the normalized clubness was > 1 and at cut-offs > 10% the diverse clubness increased disproportionally, we used the highest 10% strengths and PCs within individual networks (i.e., 30 nodes) as rich or diverse clubs.

Clubness definition

The size of the rich and diverse clubs were computed by first assessing normalized clubness coefficient in a range of cut-off values. As the graphs in our sample were fully weighted so that the degree of every node n is theoretically $n - 1$, we used a range of percentages to define the clubness cut-off, as shown in Figure A.2. Nodes that most frequently belonged to either club are presented in Figure A.3; cortical node names correspond to the Schaefer atlas.[23]

The rich and diverse club coefficients were calculated as follows, in line with earlier studies.[21, 22] First, each node's strength was calculated by summing the edge strength with all other nodes and participation coefficient was calculated with the Brain Connectivity Toolbox function *participation_coef.m*, using the community structure as calculated with the generalized Louvain method. Nodes were sorted on strength or participation coefficient and the highest values were

extracted using a range of cut-offs from 30% to 5% (corresponding to a range of 90 to 15 nodes), to define rich and diverse clubs with different sizes, respectively.

For these clubs, the clubness coefficient θ was calculated by dividing total edge strength by total possible edge strength:

$$\theta = \frac{e}{n\,(n-1)\,/\,2}$$

where e was the sum of edge club's edge strengths and n was the number of nodes. Similarly, this process was repeated to compute random network clubness θ_{rand} for 1000 random networks that were computed per individual preserving the degree-, weight-, and strength-distributions, using the Brain Connectivity Toolbox function *null_model_und_sign*. Lastly, normalized clubness coefficient (θ_{norm}) was computed:

$$\theta_{rand} = \frac{\theta}{\theta_{norm}}$$

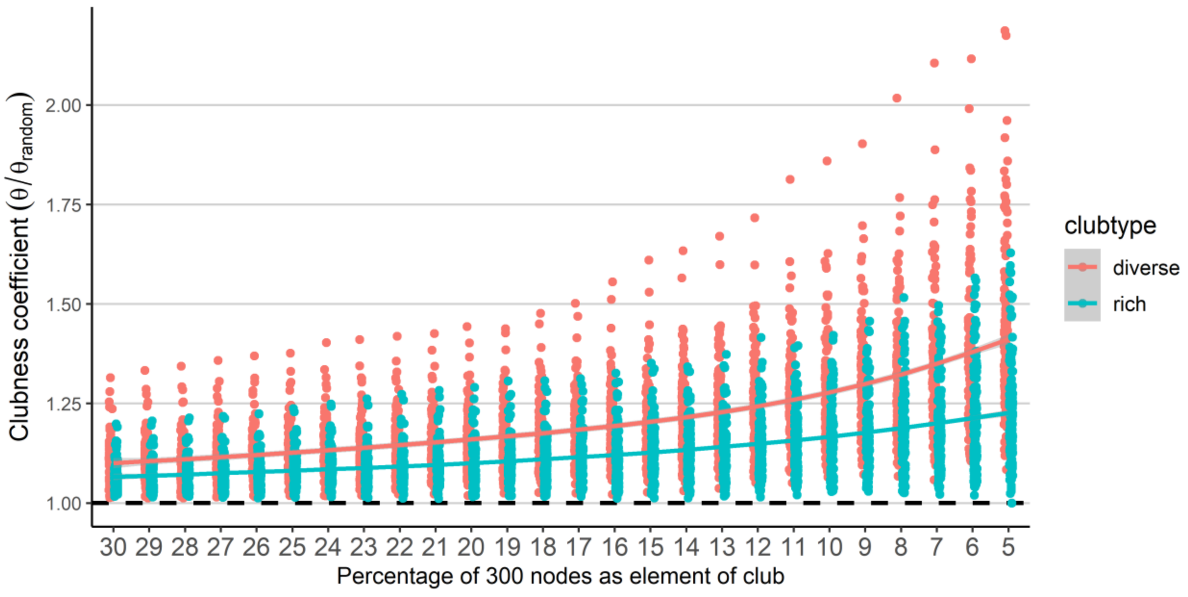

Figure A.2 Normalized rich and diverse club coefficient for a range of club size cut-offs.

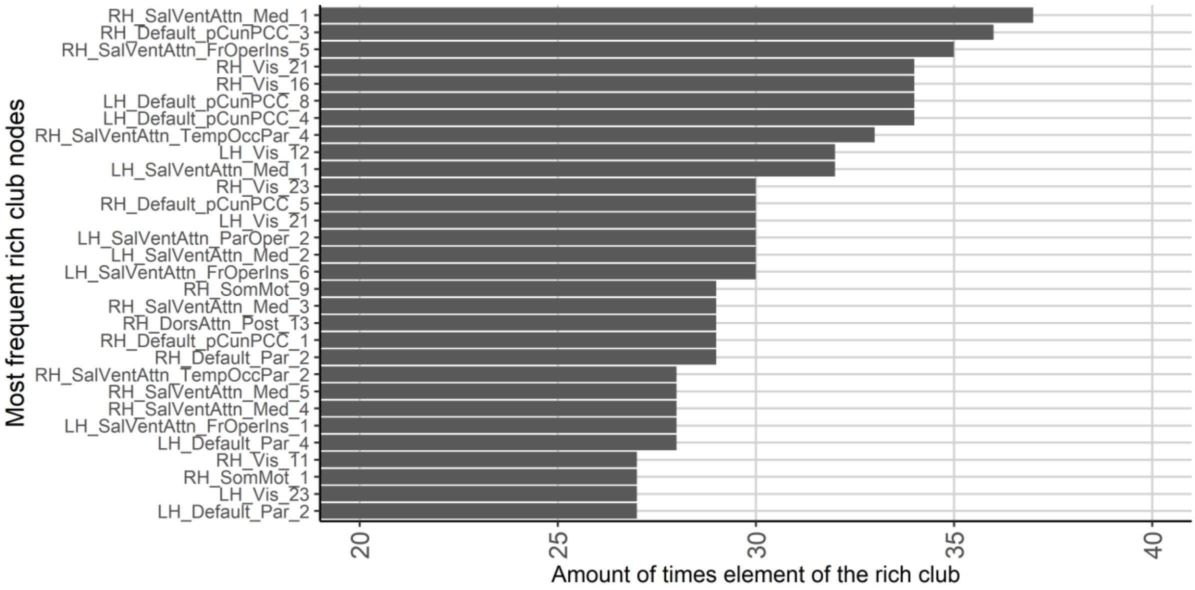

Figure A.3a Network nodes that most frequently were element of the rich club.

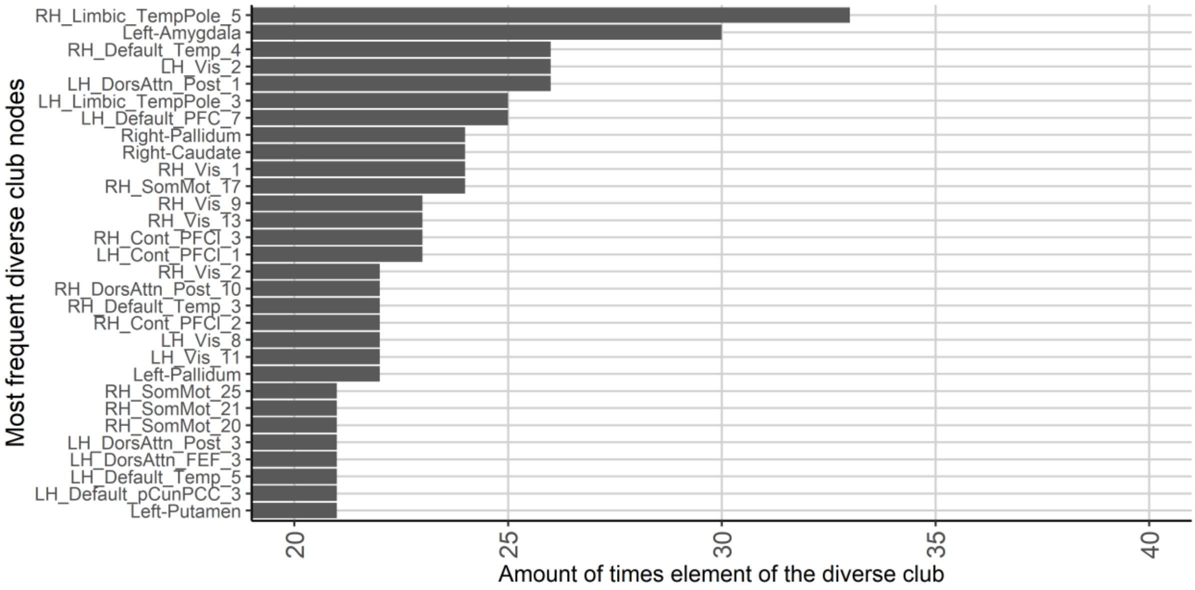

Figure A.3b Network nodes that most frequently were element of the diverse club.

Appendix A.5 – Comparison between fMRI and non-fMRI sample

Table A.2 Comparison between sample included in fMRI analyses and sample not included of the full COGTIPS intention-to-treat sample (n = 136).

	fMRI sample (n=71)	Non-fMRI sample (n=65)	p-value
**Sex (N (%))**			.861
**Male**	42 (69%)	40 (51%)	
**Female**	29 (31%)	25 (49%)	
**Age (years)**	63.5 (7.3)	62.2 (7.8)	.337
**Education (years)**	16.1 (4.0)	16.1 (3.9)	.919
**Education classification (N (%))[a]**			.949
**3**	1 (1.4%)	1 (1.5%)	
**4**	5 (7%)	3 (4.6%)	
**5**	17 (23.9%)	16 (24.6%)	
**6**	27 (38%)	28 (43.1%)	
**7**	21 (29.6%)	17 (26.2%)	
**Disease duration (years, median [range])**	4 [0-16]	7 [1-26]	**.008**
**UPDRS-III**	20.0 (9.1)	21.1 (8.7)	.482
**Hoehn & Yahr stage (N (%))**			.733
**1**	5 (7%)	4 (6.2%)	
**1.5**	5 (7%)	4 (6.2%)	
**2**	32 (45.1%)	30 (46.2%)	
**2.5**	20 (28.2%)	16 (24.6%)	
**3**	9 (12.7%)	11 (16.9%)	
**LEDD (median [range])**	640 [0-1790]	720 [0-2100]	.754
**MoCA**	26.4 (2.1)	25.8 (2.2)	.136
**Global cognitive function classification (N (%))**			.079
**Normal cognition**	19 (26.8%)	9 (13.8%)	
**Single-domain MCI**	11 (15.5%)	5 (7.7%)	
**Multi-domain MCI**	30 (42.3%)	39 (60.0%)	
**PD dementia**	11 (15.5%)	12 (18.5%)	
**BDI**	8.2 (4.0)	7.9 (4.1)	.663
**PD-CFRS (median [range])**	7.2 [3.3-22]	8.7 [3.3-19]	.944

Data are mean (SD) unless otherwise specified. [a]According to Verhage education classification.[29]
Abbreviations: BDI = Beck Depression Inventory; PD-CFRS = Parkinson's Disease – Cognitive Functional Rating Scale; LEDD = Levodopa equivalent daily dosage; MCI = mild cognitive impairment; MoCA = Montreal Cognitive Assessment; UPDRS = Unified Parkinson's Disease Rating Scale.

Appendix A.6 – Cognitive performance of the study sample

Table A.3 Neuropsychological function based on healthy norms (intention-to-treat sample).

Baseline	Total	Active control	Cognitive training	*P* value*
SCWT card I (N=71)	35.75 (10.64)	35.68 (12.89)	35.81 (8.25)	0.958
SCWT card II (N=71)	40.89 (10.70)	40.59 (11.66)	41.16 (9.89)	0.823
SCWT card III (N=71)	45.06 (11.25)	46.00 (12.42)	44.19 (10.14)	0.502
SCWT interference score (N=71)	52.31 (9.94)	53.74 (9.89)	51.00 (9.94)	0.250
Letter fluency (N=71)	48.82 (11.67)	48.76 (12.76)	48.86 (10.74)	0.971
Category fluency (N=70)	48.09 (10.51)	48.38 (11.22)	47.81 (9.94)	0.820
RCFT (N=71)	49.08 (15.47)	47.65 (16.33)	50.41 (14.74)	0.457
RAVLT direct recall (N=71)	43.92 (12.51)	44.21 (13.67)	43.65 (11.52)	0.853
RAVLT delayed recall (N=71)	46.28 (12.47)	46.18 (13.28)	46.38 (11.85)	0.946
RAVLT delayed recall corrected for direct recall score (N=71)	50.55 (10.58)	50.03 (10.44)	51.03 (10.84)	0.695
Digit span forward score (N=71)	33.59 (7.28)	32.88 (8.13)	34.24 (6.44)	0.435
Digit span backward score (N=71)	41.31 (7.30)	41.50 (7.37)	41.14 (7.34)	0.835
LLT learning curve (N=70)[24]	49.67 (7.83)	49.29 (6.67)	50.03 (8.87)	0.698
LLT direct recall (N=69)[24]	53.57 (7.35)	53.53 (8.49)	53.59 (6.33)	0.972
LLT delayed recall (N=70)[24]	48.82 (11.67)	48.76 (12.76)	48.86 (10.74)	0.971
BNT (N=71)[25]	53.99 (8.50)	53.65 (9.75)	54.30 (7.27)	0.750
BVFDT (N=71)[26]	48.17 (8.85)	47.82 (9.60)	48.49 (8.21)	0.755

*T-test. Norm scores are "T-scores" (M: 50, SD: 10) based on norms provided in Schmand and colleagues, unless otherwise specified.[27] No clinical norm data were available for the computerized Tower of London test. *Abbreviations:* BNT – Boston Naming Test; BVFDT – Benton Visual Form Discrimination Test; LLT – Location Learning Test; RAVLT – Rey Auditory Verbal Learning Test; RCFT – Rey Complex Figure Test; SCWT – Stroop Color Word Test.

Appendix A.7 – Summary of behavioral results in the fMRI and full study sample

The CT vs. AC group differences after training in the fMRI sample are shown in Table A.4. The full study sample group differences are shown in Table A.5. The estimates show that there were no significant positive training effects on the ToL reaction time and SCWT in the current subsample, in contrast with the full study sample. In the current subsample there were trend-significant to significant group differences on the ToL accuracy in favor of the AC, while these were not present in the full study sample.

Regarding the full study sample, there was no group difference on ToL accuracy after training across all task-loads S1-S5 or on any individual task-load. Multivariate analysis of the ToL reaction times across task loads S1-S5 (N=130) showed, however, group differences after training with a significant effect on ToL reaction time load S4. Estimates of the other ToL task-loads indicated numerically similar, but non-significant, positive effects of CT compared with the AC. Univariate analyses of individual neuropsychological test outcomes additionally showed estimates suggesting improvement in the CT group on the Stroop Color-Word Test card II and III that, however, did not survive correction for multiple comparisons.

Table A.4 Behavioral results in fMRI sample.

	Baseline		T1		Crude model			Adjusted model[a]		
	AC	CT	AC	CT	B [SE]	95% CI	p-value	B [SE]	95% CI	p-value
**ToL overall accuracy (%)[b]**	83.1 (7.6)	80.6 (9.0)	85.4 (8.3)	81.8 (11.9)	*-0.267 [0.153]*	*-0.573 to 0.039*	*0.086*	**-0.306 [0.147]**	**-0.599 to -0.013**	**0.041**
**S1**	96.6 (4.1)	95.4 (5.8)	97.3 (4.4)	95.3 (5.1)	-0.374 [0.235]	-0.837 to 0.090	0.114	-0.412 [0.231]	-0.867 to 0.043	0.076
**S2**	92.2 (9.7)	89.4 (10.2)	94.5 (6.3)	90.4 (13.4)	-0.311 [0.236]	-0.775 to 0.153	0.188	-0.351 [0.231]	-0.806 to 0.104	0.130
**S3**	89.2 (8.9)	85.7 (13.0)	90.6 (8.7)	85.8 (16.2)	-0.327 [0.236]	-0.791 to 0.137	0.167	-0.367 [0.231]	-0.822 to 0.088	0.113
**S4**	77.0 (12.2)	76.2 (14.1)	80.5 (15.1)	76.0 (17.4)	-0.258 [0.235]	-0.721 to 0.204	0.272	-0.294 [0.231]	-0.748 to 0.160	0.204
**S5**	60.6 (22.3)	56.3 (18.7)	64.2 (23.4)	61.4 (24.2)	-0.064 [0.235]	-0.527 to 0.399	0.786	-0.103 [0.231]	-0.557 to 0.352	0.657
**ToL overall reaction time(s)[c,d]**	12.8 (3.1)	12.2 (3.2)	12.3 (3.1)	11.5 (2.8)	-0.148 [0.120]	-0.388 to 0.093	0.224	-0.119 [0.122]	-0.364 to 0.126	0.335
**S1**	6.4 (1.7)	5.9 (1.9)	5.9 (2.2)	5.2 (1.5)	-0.222 [0.164]	-0.546 to 0.101	0.176	-0.197 [0.165]	-0.523 to 0.128	0.234
**S2**	8.2 (2.7)	8.1 (2.9)	7.4 (1.7)	7.1 (2.4)	-0.116 [0.163]	-0.438 to 0.207	0.480	-0.084 [0.165]	-0.409 to 0.241	0.611
**S3**	10.8 (2.9)	10.7 (3.3)	10.7 (3.6)	9.8 (3.5)	-0.209 [0.163]	-0.531 to 0.113	0.202	-0.178 [0.165]	-0.503 to 0.148	0.283
**S4**	15.7 (4.3)	15.3 (5.0)	15.5 (4.9)	14.1 (5.2)	-0.261 [0.163]	-0.584 to 0.061	0.111	-0.232 [0.165]	-0.557 to 0.093	0.161
**S5**	22.8 (5.8)	21.2 (4.8)	21.8 (4.5)	21.3 (4.1)	0.077 [0.165]	-0.248 to 0.402	0.641	0.102 [0.166]	-0.226 to 0.429	0.541
**Stroop Color Word Test**										
1 (word reading speed)	56.0 (16.8)	53.8 (9.4)	52.2 (12.7)	50.8 (8.2)	-0.203 [1.797]	-3.787 to 3.380	0.910	0.425 [1.732]	-3.030 to 3.879	0.807
2 (color naming speed)	67.3 (15.1)	65.4 (12.4)	67.3 (18.6)	64.4 (16.5)	-0.994 [2.422]	-5.824 to 3.836	0.683	-1.423 [2.413]	-6.234 to 3.388	0.557
3 (color-word interference)	110.4 (42.7)	116.0 (55.3)	104.4 (36.6)	99.7 (24.8)	-7.315 [4.827]	-16.942 to 2.313	0.134	-7.442 [4.907]	-17.228 to 2.345	0.134

[a]Corrected for age, sex and education in years; [b]Positive estimates indicate effects in favor of CT; [c]Negative estimates indicate effects in favor of CT; [d]Reaction time of correct responses.

Table A.5 Group differences from the linear mixed-model analyses on the ToL accuracy, ToL reaction time and Stroop Color Word Test for the crude and adjusted analysis models.

	Baseline		T1		Group difference (crude model)			Group difference (adjusted model)[a]		
	Active control M (SD)	Cognitive training M (SD)	Active control M (SD)	Cognitive training M (SD)	B [SE]	95% CI	*P* value	B [SE]	95% CI	*P* value
**Overall ToL accuracy (%)**[b]	81.5 (9.1)	79.9 (11.9)	84.9 (8.1)	83.1 (11.6)	-0.12 [0.10]	-0.33 to 0.09	0.255	-0.13 [0.10]	-0.33 to 0.08	0.229
S1	95.7 (6.8)	95.1 (7.8)	97.0 (4.9)	95.4 (7.4)	-0.23 [0.16]	-0.54 to 0.08	0.144	-0.23 [0.16]	-0.54 to 0.07	0.133
S2	91.4 (9.7)	89.4 (11.5)	92.7 (8.4)	89.4 (14.0)	-0.22 [0.16]	-0.53 to 0.09	0.160	-0.23 [0.16]	-0.53 to 0.08	0.144
S3	86.4 (13.7)	85.2 (16.2)	88.9 (9.1)	88.4 (13.5)	-0.02 [0.16]	-0.33 to 0.28	0.881	-0.03 [0.16]	-0.34 to 0.28	0.852
S4	74.9 (14.8)	73.4 (17.9)	80.8 (13.9)	77.7 (17.8)	-0.16 [0.16]	-0.46 to 0.15	0.315	-0.16 [0.16]	-0.47 to 0.14	0.296
S5	59.2 (20.5)	56.3 (21.0)	64.9 (21.7)	64.6 (22.3)	0.03 [0.16]	-0.28 to 0.34	0.840	0.02 [0.16]	-0.28 to 0.33	0.874
**Overall ToL reaction time (s)**[c,d]	12.8 (2.8)	12.5 (3.2)	12.3 (3.1)	11.4 (2.8)	-0.19 [0.10]	-0.39 to 0.00	0.052	-0.14 [0.10]	-0.34 to 0.06	0.165
S1	6.4 (2.0)	6.0 (1.8)	6.0 (2.3)	5.4 (1.7)	-0.22 [0.13]	-0.47 to 0.03	0.086	-0.17 [0.13]	-0.42 to 0.08	0.191
S2	8.3 (2.6)	8.2 (2.9)	7.5 (2.2)	7.1 (2.4)	-0.14 [0.13]	-0.40 to 0.11	0.258	-0.09 [0.13]	-0.34 to 0.16	0.489
S3	11.1 (2.7)	11.1 (3.5)	10.7 (3.4)	10.0 (3.5)	-0.19 [0.13]	-0.44 to 0.06	0.135	-0.14 [0.13]	-0.39 to 0.12	0.292
S4	15.7 (3.9)	15.5 (4.9)	15.3 (4.8)	13.8 (4.6)	-0.28 [0.13]	-0.53 to -0.03	0.030	-0.22 [0.13]	-0.47 to 0.03	0.083
S5	22.2 (4.9)	21.6 (4.9)	21.7 (4.5)	20.8 (4.6)	-0.14 [0.13]	-0.39 to 0.12	0.289	-0.08 [0.13]	-0.33 to 0.17	0.532
**Stroop Color Word Test**										
1 (word reading speed)	56.3 (15.1)	54.1 (10.7)	54.3 (13.2)	50.8 (9.1)	-2.17 [1.35]	-4.85 to 0.51	0.574[e] 0.111	-1.57 [1.35]	-4.25 to 1.07	0.700[e] 0.239
2 (color naming speed)	68.0 (13.8)	68.2 (18)	68.6 (16.4)	64.8 (15.8)	-3.95 [1.83]	-7.57 to -0.34	0.331[e] 0.032	-3.64 [1.82]	-7.25 to -0.03	0.501[e] 0.048
3 (color-word interference)	106.9 (33.9)	114.9 (49.3)	103.4 (30.7)	101.7 (28.6)	-6.02 [3.33]	-12.61 to 0.56	0.453[e] 0.073	-5.65 [3.38]	-12.33 to 1.03	0.501[e] 0.097

[a]Corrected for age, sex and education in years; [b]Positive estimates indicate effects in favor of CT; [c]Negative estimates indicate effects in favor of CT; [d]Reaction time of correct responses; [e]FDR-corrected p-values.

Appendix A.8 – Analysis of graph outcomes calculated with Pearson correlation-based connectivity matrices (instead of wavelet coherence-based)

Graph outcomes calculated with Pearson correlation-based connectivity matrices showed high correspondence with the wavelet-coherence based graph outcomes, as shown below (Table A.6).

Table A.6 Correlation of global and subnetwork graph measures calculated by the wavelet coherence method with those calculated by the Pearson correlation method.

Variable	r	Variable	r
Global efficiency	.768	FPN – Global efficiency	.876
Participation coefficient	.548	FPN – Betweenness centrality	.553
Modularity	.455	FPN – Clustering coefficient	.549
SN – Global efficiency	.937	DMN – Global efficiency	.747
SN – Betweenness centrality	.672	DMN – Betweenness centrality	.559
SN – Clustering coefficient	.712	DMN – Clustering coefficient	.403

Table A.7 Group differences, corrected for baseline value, on primary neuroimaging outcomes calculated on the basis of Pearson correlation connectivity matrices.

		**Graph outcomes**					
		**Crude models**			**Adjusted models[a]**		
		B [SE]	95% CI	p-value	B [SE]	95% CI	p-value
	**GE**	0.004 [0.004]	-0.004 to 0.013	0.301	0.003 [0.004]	-0.005 to 0.011	0.484
**Global**	**Q**	-0.006 [0.004]	-0.014 to 0.003	0.178	-0.008 [0.004]	-0.016 to 0.000	*0.060*
	**PC**	-0.002 [0.010]	-0.022 to 0.018	0.821	0.002 [0.010]	-0.018 to 0.022	0.816
	**GE**	0.002 [0.008]	-0.015 to 0.018	0.824	0.000 [0.008]	-0.017 to 0.016	0.964
**FPN**	**CC[b]**	5.113 [3.235]	-1.338 to 11.564	0.118	4.503 [3.270]	-2.018 to 11.024	0.173
	**BC[b]**	0.001 [0.193]	-0.384 to 0.386	0.995	0.058 [0.193]	-0.328 to 0.443	0.766
	**GE**	0.003 [0.008]	-0.013 to 0.018	0.738	0.000 [0.008]	-0.016 to 0.015	0.959
**DMN**	**CC[b]**	5.207 [3.557]	-1.885 to 12.300	0.148	4.159 [3.518]	-2.856 to 11.174	0.241
	**BC[b]**	-0.106 [0.130]	-0.365 to 0.154	0.421	-0.119 [0.129]	-0.376 to 0.138	0.359
	**GE**	0.003 [0.011]	-0.020 to 0.025	0.819	-0.003 [0.011]	-0.025 to 0.020	0.808
**SN**	**CC[b]**	3.972 [4.260]	-4.522 to 12.465	0.354	2.474 [4.242]	-5.984 to 10.931	0.562
	BC[b]	-0.036 [0.184]	-0.404 to 0.331	0.844	-0.042 [0.187]	-0.416 to 0.331	0.822

[a]Corrected for age, sex, education in years and, for between-network connectivity analyses, framewise displacement.
[b]Statistics multiplied by 10^3 because of small values. Abbreviations: BC – Betweenness centrality; CC – Clustering coefficient; DMN – Default mode network; FPN – Frontoparietal network; GE – Global efficiency; PC – Participation coefficient; Q – Modularity; SN – Salience network.

Table A.8 Univariate mixed-model analysis of group differences on global graph outcomes per sub-group based on cognitive status calculated on the basis of Pearson correlation connectivity matrices.

	**PD-NC (n=19)**			**PD-MCI (n=41)**			**PD-D (n=11)**		
	**B [SE]**	**95% CI**	**p-value**	**B [SE]**	**95% CI**	**p-value**	**B [SE]**	**95% CI**	**p-value**
**GE**	0.009 [0.008]	-0.006 to 0.025	0.230	0.002 [0.005]	-0.009 to 0.012	0.740	-0.010 [0.011]	-0.033 to 0.012	0.356
**Q**	0.000 [0.008]	-0.015 to 0.016	0.960	-0.010 [0.005]	-0.021 to 0.001	*0.068*	0.000 [0.012]	-0.024 to 0.024	0.998
**PC**	-0.041 [0.017]	-0.076 to -0.007	**0.019**	0.022 [0.012]	-0.002 to 0.045	*0.072*	-0.007 [0.026]	-0.058 to 0.045	0.792

Based on 3 comparisons per sub-group, with mean r = .443 between the graph outcomes, corrected α = .027.
Abbreviations: GE – Global efficiency; Q – Modularity; PC – Participation coefficient; PD-NC – Normal cognition; PD-MCI – Mild cognitive impairment; PD-D – Dementia.

Appendix A.9 – Repeated-measures correlation analyses

Table A.9a Repeated measures correlation analyses per group of the association between change on neuropsychological test performance (left-most column) and change in between-network connectivity and network topological measures.

		Active control			Cognitive training		
		r (df)	95% CI	p-value	r (df)	95% CI	p-value
ToL Accuracy	SN - DMN	0.201 (31)	0.332 to 0.678	0.262	0.048 (35)	-0.129 to 0.202	0.778
	SN - FPN	-0.055 (31)	0.318 to 0.608	0.760	0.237 (35)	-0.137 to 0.162	0.158
	FPN - DMN	-0.158 (31)	0.119 to 0.573	0.380	-0.029 (35)	-0.178 to 0.217	0.863
	GE	0.042 (31)	0.147 to 0.550	0.816	-0.202 (35)	-0.218 to 0.202	0.230
	PC	0.055 (31)	-0.092 to 0.286	0.760	0.137 (35)	-0.154 to 0.243	0.418
	Q	0.103 (31)	-0.012 to 0.373	0.569	0.061 (35)	-0.151 to 0.119	0.722
	SN - Efficiency	0.208 (31)	0.091 to 0.503	0.246	0.003 (35)	-0.130 to 0.171	0.984
	SN - BC	0.094 (31)	0.056 to 0.477	0.603	0.048 (35)	-0.285 to 0.074	0.776
	SN - CC	0.186 (31)	0.203 to 0.526	0.301	-0.155 (35)	-0.178 to 0.197	0.359
	FPN - Efficiency	0.082 (31)	0.080 to 0.521	0.652	0.116 (35)	0.010 to 0.315	0.493
	FPN - BC	-0.097 (31)	-0.245 to 0.362	0.591	0.225 (35)	-0.218 to 0.105	0.181
	FPN - CC	0.050 (31)	0.116 to 0.559	0.784	-0.118 (35)	-0.145 to 0.308	0.487
	DMN - Efficiency	0.105 (31)	-0.264 to 0.347	0.562	-0.103 (35)	-0.368 to 0.130	0.543
	DMN - BC	0.051 (31)	-0.307 to 0.178	0.777	0.298 (35)	-0.092 to 0.338	0.073
	DMN - CC	0.143 (31)	-0.009 to 0.364	0.428	-0.190 (35)	-0.201 to 0.235	0.260
ToL Reaction Time	SN - DMN	-0.070 (31)	-0.497 to -0.079	0.699	0.292 (34)	-0.358 to -0.010	0.084
	SN - FPN	-0.093 (31)	-0.353 to 0.019	0.608	0.086 (34)	-0.301 to 0.123	0.619
	FPN - DMN	0.031 (31)	-0.534 to -0.168	0.865	0.077 (34)	-0.383 to 0.006	0.653
	GE	-0.005 (31)	-0.237 to 0.307	0.976	-0.219 (34)	-0.529 to -0.176	0.199
	PC	0.287 (31)	0.074 to 0.337	0.105	0.205 (34)	-0.082 to 0.457	0.229
	Q	-0.367 (31)	-0.472 to -0.134	0.036	-0.002 (34)	-0.297 to 0.062	0.990
	SN - Efficiency	-0.147 (31)	-0.247 to 0.114	0.413	-0.015 (34)	-0.408 to 0.095	0.929
	SN - BC	-0.172 (31)	-0.445 to 0.032	0.337	0.261 (34)	0.033 to 0.399	0.124
	SN - CC	-0.042 (31)	-0.224 to 0.267	0.815	-0.155 (34)	-0.391 to -0.058	0.368
	FPN - Efficiency	0.245 (31)	-0.306 to 0.076	0.170	-0.056 (34)	-0.572 to -0.066	0.747
	FPN - BC	0.155 (31)	-0.486 to -0.081	0.388	0.020 (34)	-0.254 to 0.212	0.909
	FPN - CC	0.210 (31)	-0.202 to 0.303	0.241	-0.312 (34)	-0.553 to -0.149	0.064
	DMN - Efficiency	-0.283 (31)	-0.443 to 0.104	0.111	-0.247 (34)	-0.236 to 0.201	0.146
	DMN - BC	-0.329 (31)	-0.440 to -0.066	0.062	0.181 (34)	-0.122 to 0.207	0.292
	DMN - CC	-0.013 (31)	-0.200 to 0.315	0.945	-0.346 (34)	-0.497 to -0.079	0.039

Based on 15 comparisons per neuropsychological test, with mean r = .264, corrected α = .007.
Abbreviations: BC – Betweenness centrality; CC – Clustering coefficient; DMN – Default mode network; FPN – Frontoparietal network; GE – Global efficiency; PC – Participation coefficient; Q – Modularity; SN – Salience network; ToL – Tower of London task

Table A.9b Repeated measures correlation analyses per group of the association between change on neuropsychological test performance (left-most column) and change in between-network connectivity and network topological measures.

		Active control			Cognitive training		
		**r (df)**	**95% CI**	**p-value**	**r (df)**	**95% CI**	**p-value**
	**SN - DMN**	0.049 (33)	-0.215 to 0.185	0.779	0.271 (36)	-0.369 to -0.043	0.100
	**SN - FPN**	0.347 (33)	-0.124 to 0.218	0.041	0.107 (36)	-0.218 to 0.157	0.523
	**FPN - DMN**	0.002 (33)	-0.247 to 0.109	0.992	0.092 (36)	-0.239 to 0.096	0.584
	**GE**	0.351 (33)	-0.010 to 0.417	0.039	0.025 (36)	-0.238 to 0.083	0.881
	**PC**	0.043 (33)	-0.190 to 0.260	0.804	-0.003 (36)	-0.051 to 0.292	0.986
	**Q**	-0.211 (33)	-0.076 to 0.265	0.224	0.012 (36)	-0.356 to -0.073	0.942
	**SN - Efficiency**	0.219 (33)	0.059 to 0.552	0.206	0.089 (36)	-0.341 to -0.022	0.593
**SCWT 1**	**SN - BC**	-0.063 (33)	-0.133 to 0.256	0.718	0.105 (36)	-0.303 to 0.238	0.529
	**SN - CC**	0.400 (33)	0.088 to 0.572	0.017	0.045 (36)	-0.171 to 0.063	0.789
	**FPN - Efficiency**	0.118 (33)	-0.367 to 0.024	0.500	-0.025 (36)	-0.112 to 0.241	0.880
	**FPN - BC**	-0.138 (33)	-0.568 to -0.279	0.428	-0.183 (36)	-0.053 to 0.370	0.272
	**FPN - CC**	0.399 (33)	-0.135 to 0.361	0.018	-0.063 (36)	-0.082 to 0.293	0.708
	**DMN - Efficiency**	0.013 (33)	-0.561 to -0.109	0.942	-0.101 (36)	-0.183 to 0.204	0.547
	**DMN - BC**	-0.367 (33)	-0.617 to -0.268	0.030	0.130 (36)	-0.231 to 0.214	0.438
	**DMN - CC**	0.353 (33)	-0.244 to 0.173	0.037	-0.128 (36)	-0.217 to 0.229	0.443
	**SN - DMN**	-0.029 (33)	-0.349 to -0.006	0.870	0.224 (36)	-0.541 to -0.065	0.176
	**SN - FPN**	-0.287 (33)	-0.522 to -0.080	0.095	0.205 (36)	-0.249 to 0.223	0.216
	**FPN - DMN**	-0.200 (33)	-0.361 to -0.028	0.249	0.139 (36)	-0.295 to 0.185	0.405
	**GE**	-0.315 (33)	-0.404 to 0.433	0.065	0.079 (36)	-0.292 to 0.185	0.636
	**PC**	0.323 (33)	-0.123 to 0.240	0.058	0.018 (36)	-0.209 to 0.147	0.916
	**Q**	0.005 (33)	-0.224 to 0.301	0.976	-0.009 (36)	-0.277 to 0.077	0.959
	**SN - Efficiency**	-0.205 (33)	-0.153 to 0.331	0.238	0.019 (36)	-0.356 to 0.065	0.909
**SCWT 2**	**SN - BC**	0.366 (33)	-0.157 to 0.421	0.031	0.202 (36)	-0.231 to 0.257	0.224
	**SN - CC**	-0.317 (33)	-0.332 to 0.426	0.063	0.013 (36)	-0.248 to 0.148	0.938
	**FPN - Efficiency**	-0.174 (33)	-0.542 to 0.022	0.318	0.063 (36)	-0.121 to 0.384	0.707
	**FPN - BC**	-0.185 (33)	-0.473 to -0.204	0.288	0.133 (36)	-0.012 to 0.379	0.426
	**FPN - CC**	-0.330 (33)	-0.533 to 0.327	0.053	0.000 (36)	-0.117 to 0.334	0.999
	**DMN - Efficiency**	-0.171 (33)	-0.555 to -0.190	0.325	-0.078 (36)	-0.255 to 0.162	0.641
	**DMN - BC**	0.121 (33)	-0.539 to 0.101	0.488	0.306 (36)	-0.309 to 0.039	0.062
	**DMN - CC**	-0.308 (33)	-0.613 to 0.111	0.072	-0.060 (36)	-0.212 to 0.246	0.721
	**SN - DMN**	-0.137 (33)	-0.226 to 0.208	0.433	0.124 (36)	-0.375 to -0.046	0.458
	**SN - FPN**	-0.106 (33)	-0.417 to 0.007	0.546	0.259 (36)	-0.222 to 0.484	0.116
	**FPN - DMN**	0.094 (33)	-0.233 to 0.274	0.593	0.081 (36)	-0.317 to 0.120	0.629
	**GE**	0.021 (33)	-0.369 to 0.428	0.905	-0.011 (36)	-0.191 to 0.190	0.948
	**PC**	-0.230 (33)	-0.512 to -0.088	0.183	-0.084 (36)	-0.342 to 0.139	0.618
	**Q**	0.260 (33)	-0.010 to 0.353	0.131	0.238 (36)	-0.185 to 0.262	0.150
	**SN - Efficiency**	0.207 (33)	-0.130 to 0.359	0.233	0.079 (36)	-0.197 to 0.253	0.638
**SCWT 3**	**SN - BC**	0.129 (33)	-0.158 to 0.373	0.460	0.112 (36)	-0.068 to 0.356	0.505
	**SN - CC**	0.059 (33)	-0.202 to 0.406	0.737	0.013 (36)	-0.187 to 0.187	0.938
	**FPN - Efficiency**	-0.042 (33)	-0.420 to 0.086	0.812	0.129 (36)	-0.036 to 0.392	0.439
	**FPN - BC**	0.015 (33)	-0.439 to 0.066	0.930	-0.062 (36)	-0.097 to 0.337	0.710
	**FPN - CC**	-0.038 (33)	-0.347 to 0.262	0.828	-0.009 (36)	-0.126 to 0.238	0.956
	**DMN - Efficiency**	0.233 (33)	-0.437 to 0.209	0.179	0.053 (36)	-0.190 to 0.321	0.754
	**DMN - BC**	0.162 (33)	-0.377 to 0.131	0.354	-0.093 (36)	-0.328 to 0.118	0.579
	**DMN - CC**	0.013 (33)	-0.381 to 0.182	0.941	-0.037 (36)	-0.225 to 0.273	0.826

Based on 15 comparisons per neuropsychological test, with mean r = .264, corrected α = .007.

Abbreviations: BC – Betweenness centrality; CC – Clustering coefficient; DMN – Default mode network; FPN – Frontoparietal network; GE – Global efficiency; PC – Participation coefficient; Q – Modularity; SCWT – Stroop Color Word Task; SN – Salience network.

Appendix A.10 - Effects of CT grouped by cognitive status

Table A.10a Univariate mixed-model analysis of group differences on between-network connectivity per sub-group based on cognitive status.

	PD-NC (n=19)			PD-MCI (n=41)			PD-D (n=11)		
	**B [SE]**	**95% CI**	**p-value**	**B [SE]**	**95% CI**	**p-value**	**B [SE]**	**95% CI**	**p-value**
**SN-FPN**	0.020 [0.026]	-0.032 to 0.073	0.442	0.000 [0.018]	-0.036 to 0.035	0.988	0.039 [0.038]	-0.037 to 0.116	0.310
**SN-DMN**	-0.002 [0.027]	-0.056 to 0.052	0.944	0.002 [0.019]	-0.036 to 0.039	0.927	0.017 [0.039]	-0.061 to 0.095	0.665
**FPN-DMN**	0.035 [0.027]	-0.018 to 0.088	0.191	-0.023 [0.018]	-0.059 to 0.013	0.212	0.041 [0.039]	-0.037 to 0.119	0.301

Based on 3 comparisons per sub-group, with mean r = .544 between the connectivity outcomes, corrected α = .031.
Abbreviations: DMN – Default mode network; FPN – Frontoparietal network; PD-NC – Normal cognition; PD-MCI – Mild cognitive impairment; PD-D – Dementia; SN – Salience network.

Table A.10b Univariate mixed-model analysis of group differences on global graph outcomes per sub-group based on cognitive status.

	PD-NC (n=19)			PD-MCI (n=41)			PD-D (n=11)		
	**B [SE]**	**95% CI**	**p-value**	**B [SE]**	**95% CI**	**p-value**	**B [SE]**	**95% CI**	**p-value**
**GE**	0.003 [0.006]	-0.008 to 0.014	0.607	-0.001 [0.004]	-0.009 to 0.006	0.750	-0.003 [0.008]	-0.019 to 0.013	0.709
**Q**	0.008 [0.004]	0.001 to 0.016	*0.033*	-0.003 [0.003]	-0.008 to 0.002	0.269	-0.002 [0.006]	-0.013 to 0.010	0.762
**PC**	-0.042 [0.015]	-0.072 to -0.012	**0.007**	0.009 [0.010]	-0.011 to 0.030	0.370	0.003 [0.022]	-0.041 to 0.048	0.881

Based on 3 comparisons per sub-group, with mean r = .443 between the graph outcomes, corrected α = .027.
Abbreviations: GE – Global efficiency; Q – Modularity; PC – Participation coefficient; PD-NC – Normal cognition; PD-MCI – Mild cognitive impairment; PD-D – Dementia.

Table A.10c Univariate mixed-model analysis of group differences on sub-network graph outcomes per sub-group based on cognitive status.

		PD-NC (n=19)			PD-MCI (n=41)			PD-D (n=11)		
		**B [SE]**	**95% CI**	**p-value**	**B [SE]**	**95% CI**	**p-value**	**B [SE]**	**95% CI**	**p-value**
	**GE**	0.004 [0.014]	-0.024 to 0.032	0.756	-0.011 [0.010]	-0.030 to 0.008	0.269	0.006 [0.021]	-0.035 to 0.047	0.762
**FPN**	**CC**	-0.003 [0.006]	-0.016 to 0.010	0.636	-0.001 [0.004]	-0.010 to 0.007	0.728	0.003 [0.009]	-0.015 to 0.022	0.729
	**BC**	0.029 [0.142]	-0.254 to 0.312	0.838	-0.016 [0.096]	-0.209 to 0.176	0.866	0.468 [0.207]	0.055 to 0.881	*0.027*
	**GE**	0.003 [0.013]	-0.023 to 0.029	0.805	-0.013 [0.009]	-0.031 to 0.004	0.132	0.024 [0.019]	-0.014 to 0.063	0.215
**DMN**	**CC**	-0.002 [0.006]	-0.015 to 0.010	0.693	-0.002 [0.004]	-0.010 to 0.007	0.677	0.007 [0.009]	-0.011 to 0.025	0.433
	**BC**	0.113 [0.111]	-0.109 to 0.335	0.315	-0.090 [0.076]	-0.241 to 0.062	0.242	0.149 [0.163]	-0.176 to 0.474	0.363
	**GE**	0.013 [0.020]	-0.027 to 0.053	0.533	-0.019 [0.014]	-0.047 to 0.008	0.157	-0.024 [0.029]	-0.082 to 0.035	0.422
**SN**	**CC**	-0.003 [0.007]	-0.016 to 0.010	0.685	-0.003 [0.004]	-0.012 to 0.006	0.477	-0.008 [0.009]	-0.027 to 0.011	0.412
	**BC**	0.210 [0.184]	-0.156 to 0.577	0.256	-0.030 [0.123]	-0.275 to 0.216	0.811	-0.286 [0.265]	-0.815 to 0.242	0.284

Based on 9 comparisons per sub-group, with mean r = .361 between the graph outcomes, corrected α = . 0.013.
Abbreviations: DMN – Default mode network; FPN – Frontoparietal network; GE – Global efficiency; Q – Modularity; PC – Participation coefficient; PD-NC – Normal cognition; PD-MCI – Mild cognitive impairment; PD-D – Dementia; SN – Salience network;.

Appendix A.11 - Nodal connectivity and topology of sub-network key regions

Table A.11 Overview of connectivity between network key regions and intrinsic brain networks that correlated with neuropsychological outcomes and were therefore selected for post-hoc analysis.

		TOL accuracy	TOL RT	TOL RT S4	MOCA	SCWT 1	SCWT 2	SCWT 3	Letter fluency	Category fluency	RAVLT direct	RAVLT recall	Digit span forward	Digit span backward	LLT	BNT
	**Functional connectivity indices**															
	dACC R - FPN	0.097	-.288*	-.252*	0.062	0.022	-0.122	0.12	-0.117	0.088	0.157	0.218	0.047	0.105	-0.193	-0.007
	medial PFC L - SN	0.046	-.279*	-.298*	0.114	-0.086	-.241*	-0.118	0.03	0.121	0.189	0.214	0.218	0.127	-0.12	0.027
	dACC L - DMN	0.122	-.321*	-.288*	0.15	0.008	-0.118	-0.079	-0.08	0.13	0.164	.236*	0.059	0.032	-0.153	0.126
	AI R - DMN	0.206	-.243*	-.263*	.242*	-0.192	-0.163	0.014	-0.025	.267*	0.148	0.171	0.02	0.045	-.270*	0.043
	dACC R - DMN	0.142	-.347*	-.279*	0.184	-0.092	-0.22	-0.022	0.024	0.202	0.186	.297*	0.097	0.141	-0.209	0.176
	dlPFC L - FPN	0.036	-0.093	-0.068	-0.045	-.281*	-.241*	0.05	-0.055	0.062	0.085	0.05	0.117	0.088	-0.083	-0.001
	dlPFC R - FPN	0.193	-0.048	-0.057	0.043	-.287*	-0.171	0.053	-0.022	0.001	-0.012	0.072	0.135	0.138	-0.184	.234*
	medial PFC L - DMN	0.062	-0.079	-0.048	-0.09	0.026	-0.183	-0.229	.330*	0.143	0.157	0.113	0.217	.252*	-0.138	-0.006
	medial PFC R - DMN	0.054	-0.025	-0.021	0.01	-0.056	-0.173	-0.085	.312*	0.084	0.181	0.112	.276*	.312*	-0.139	0.006
	**Nodal graph indices**															
Part. coefficient	AI L	-0.048	0.188	0.15	-0.143	0.131	0.096	-0.099	0.051	-0.109	-.250*	-0.209	0.099	-0.088	-0.02	0.065
	ACC L	-0.032	0.234	0.18	-0.119	0.215	0.187	-0.017	-0.014	-0.178	-.264*	-.238*	0.048	-0.139	0.008	0.048
	Medial PFC L	-0.054	0.098	0.073	-0.087	-0.095	-0.069	-.290*	0.008	-0.063	-0.206	-0.119	0.086	-0.201	-0.105	0.026
	PCC L	-0.037	0.071	0.051	-0.121	-0.048	-0.045	-.235*	0.015	-0.072	-0.101	-0.014	0.088	-0.196	-0.17	0.034
	ACC R	-0.043	0.203	0.152	-0.125	0.16	0.133	-0.033	0.014	-0.151	-.247*	-0.211	0.041	-0.133	-0.03	0.096
	iPL R	-0.05	0.096	0.063	-0.093	-0.011	-0.052	-.274*	0.117	-0.058	-0.157	-0.112	0.078	-0.134	-0.097	0.04
	PCC R	-0.036	0.043	0.023	-0.102	-0.054	-0.061	-.286*	0.048	-0.045	-0.096	0.003	0.09	-0.188	-0.15	0.053
Betw. Centrality	ACC L	.272*	0.069	0.095	-0.08	-0.059	-0.006	0.054	-0.101	-0.027	-0.144	-0.067	0.033	0.133	-0.065	0.024
	Medial PFC L	0.185	-0.041	-0.037	-0.019	0.04	-0.161	-0.212	0.115	.238*	0.032	0.027	-0.05	0.007	0.032	0.036
	iPL R	0.068	.280*	.272*	-0.206	-0.229	-.264*	-0.095	-0.028	-0.101	-.307*	-0.196	0.045	-0.06	0.192	-0.007
	dlPFC R	0.153	0.12	0.104	0	0.017	.279*	0.173	-0.094	-0.089	-0.069	0.058	-0.114	0.037	-0.115	0.049
Clustering coefficient	AI L	0.168	-0.191	-0.218	0.129	0.061	0.02	0.028	-0.16	-0.093	.246*	0.195	-0.11	-0.091	-0.05	0.006
	ACC L	.339*	-0.189	-0.23	0.117	0.072	0.036	0.145	-0.113	0.018	0.213	.237*	-0.01	0.128	-0.141	0.051
	iPL L	0.131	-0.224	-.246*	0.071	-0.058	-0.079	-0.075	0.015	-0.017	0.113	0.159	-0.071	-0.023	-0.145	0.037
	PCC L	0.012	-0.233	-.261*	0.205	0.136	0.093	0.01	0.156	0.064	.296*	.311*	-0.078	0.029	-0.035	-0.032
	AI R	0.154	-0.19	-0.185	0.112	0.07	0.062	0.139	-0.124	0.005	.273*	.270*	-0.127	0.004	-0.006	0.007
	ACC R	0.186	-0.185	-.242*	0.041	0.077	0.031	0.087	-0.101	0.004	0.197	0.193	-0.037	0.057	-0.045	0.038
	dlPFC R	0.164	0.011	-0.064	0.067	0.057	0.148	0.159	-0.093	-0.001	0.201	.254*	-0.016	0.034	-0.209	-0.009
	Medial PFC R	.242*	-.278*	-.285*	0.095	0.001	-0.108	-0.175	0.075	0.024	.344*	.290*	0.136	0.054	-0.052	0.147
	PCC R	-0.03	-.270*	-.308*	0.222	0.065	0.053	-0.084	0.094	0.098	.254*	.272*	-0.144	-0.077	-0.08	-0.032

Pearson correlation coefficients, * marks significant correlations p<.05.

Abbreviations: TOL = Tower of London, MOCA = Montreal Cognitive Assessment, SCWT = Stroop Color Word Task, RAVLT = Rey Auditory Verbal Learning Test, LLT = Location learning test, BNT = Boston naming test, ACC = Anterior cingulate cortex, FPN = Frontoparietal network, PFC = prefrontal cortex, SN = Salience network, DMN = Default mode network, AI = anterior insula, dlPFC = dorsolateral prefrontal cortex.

Table A.12 Selected sub-network key region node coordinates.

Schaefer node	Network region	MNI Coordinates		
7NetworksLH_SalVentAttn_FrOperIns_3	Dorsal anterior insula L	-33	19	8
7Networks_LH_SalVentAttn_Med_1	Caudodorsal ACC L	-6	9	41
7Networks_LH_Cont_Par_1	Inferior posterior parietal cortex L	-54	-50	45
7Networks_LH_Cont_PFCl_3	Dorsolateral PFC L	-41	41	14
7Networks_LH_Default_PFC_11	Medial PFC L	-4	50	31
7Networks_LH_Default_pCunPCC_4	PCC/Precuneus L	-5	-56	28
7Networks_RH_SalVentAttn_FrOperIns_3	Dorsal anterior insula R	38	21	4
7Networks_RH_SalVentAttn_Med_1	Caudodorsal ACC R	7	9	41
7Networks_RH_Cont_Par_2	Inferior posterior parietal cortex R	53	-42	48
7Networks_RH_Cont_PFCl_5	Dorsolateral PFC R	44	44	11
7Networks_RH_Default_PFCdPFCm_8	Medial PFC R	5	40	43
7Networks_RH_Default_pCunPCC_3	PCC/Precuneus R	5	-54	24

Abbreviations: ACC – anterior cingulate cortex; L – left; PFC – prefrontal cortex; PCC – posterior cingulate cortex; R – right.

Table A.13 Group differences on post-hoc outcomes of sub-network key region functional connectivity with cognitive networks.

	Crude model			Adjusted model*		
	**B [SE]**	**95% CI**	**p-value**	**B [SE]**	**95% CI**	**p-value**
**dACC R - FPN**	0.037 [0.019]	-0.001 to 0.075	0.054	0.041 [0.019]	0.003 to 0.079	*0.034*
**dACC L - DMN**	0.020 [0.021]	-0.022 to 0.062	0.349	0.016 [0.021]	-0.026 to 0.058	0.446
**AI R - DMN**	-0.011 [0.020]	-0.052 to 0.030	0.604	-0.011 [0.019]	-0.049 to 0.028	0.588
**dACC R - DMN**	0.020 [0.021]	-0.022 to 0.062	0.352	0.017 [0.021]	-0.025 to 0.058	0.423
**medial PFC L - DMN**	0.038 [0.029]	-0.020 to 0.097	0.196	0.034 [0.029]	-0.023 to 0.091	0.238
**medial PFC R - DMN**	-0.009 [0.028]	-0.065 to 0.047	0.749	-0.020 [0.027]	-0.074 to 0.034	0.462
**medial PFC L - SN**	-0.002 [0.029]	-0.060 to 0.056	0.937	-0.007 [0.029]	-0.064 to 0.051	0.817
**dlPFC L - FPN**	0.054 [0.022]	0.009 to 0.099	*0.018*	0.054 [0.022]	0.010 to 0.098	*0.017*
**dlPFC R - FPN**	0.022 [0.027]	-0.033 to 0.076	0.432	0.020 [0.028]	-0.035 to 0.076	0.467

Based on 9 comparisons, with mean r = .249 between the outcomes, corrected α = .010. *Corrected for age, sex, education in years and framewise displacement. *Abbreviations:* AI – anterior insula; dACC – dorsal anterior cingulate cortex; DMN – Default mode network; dlPFC – dorsolateral prefrontal cortex; FPN – Frontoparietal network; PFC – prefrontal cortex; SN – Salience network.

Table A.14 Group differences on post-hoc outcomes of sub-network key region functional connectivity with subcortical structures.

		Cortico-subcortical connectivity of sub-network key regions					
		**Crude model**			**Adjusted model***		
		**B [SE]**	**95% CI**	**p-value**	**B [SE]**	**95% CI**	**p-value**
**dACC L**	**Thalamus L**	0.036 [0.030]	-0.023 to 0.096	0.225	0.022 [0.029]	-0.036 to 0.081	0.450
	**Thalamus R**	0.072 [0.033]	0.006 to 0.138	*0.032*	0.054 [0.032]	-0.009 to 0.118	0.092
	**Caudate L**	0.076 [0.034]	0.008 to 0.145	*0.028*	0.070 [0.034]	0.002 to 0.137	*0.045*
	**Caudate R**	0.060 [0.029]	0.001 to 0.118	*0.046*	0.052 [0.029]	-0.005 to 0.110	0.075
	**Hippocampus L**	0.037 [0.035]	-0.033 to 0.107	0.299	0.037 [0.036]	-0.035 to 0.110	0.304
	**Hippocampus R**	0.032 [0.035]	-0.038 to 0.102	0.370	0.027 [0.035]	-0.043 to 0.098	0.441
**dACC R**	**Thalamus L**	0.056 [0.034]	-0.012 to 0.123	0.104	0.046 [0.034]	-0.022 to 0.113	0.182
	**Thalamus R**	0.094 [0.037]	0.021 to 0.167	*0.013*	0.085 [0.037]	0.012 to 0.158	*0.024*
	**Caudate L**	0.083 [0.035]	0.013 to 0.154	*0.021*	0.074 [0.035]	0.005 to 0.143	*0.036*
	**Caudate R**	0.094 [0.030]	0.034 to 0.154	**0.003**	0.088 [0.029]	0.030 to 0.145	**0.004**
	**Hippocampus L**	-0.003 [0.038]	-0.080 to 0.073	0.928	0.004 [0.039]	-0.074 to 0.082	0.915
	**Hippocampus R**	0.029 [0.035]	-0.041 to 0.099	0.408	0.029 [0.035]	-0.041 to 0.099	0.414
**dlPFC L**	**Thalamus L**	0.016 [0.035]	-0.055 to 0.086	0.662	0.006 [0.035]	-0.064 to 0.076	0.871
	**Thalamus R**	0.041 [0.043]	-0.046 to 0.127	0.351	0.031 [0.043]	-0.056 to 0.117	0.481
	**Caudate L**	0.060 [0.032]	-0.005 to 0.124	0.071	0.045 [0.031]	-0.018 to 0.107	0.159
	**Caudate R**	0.034 [0.038]	-0.043 to 0.110	0.383	0.026 [0.039]	-0.052 to 0.103	0.513
	**Hippocampus L**	-0.036 [0.036]	-0.108 to 0.037	0.328	-0.039 [0.035]	-0.109 to 0.030	0.264
	**Hippocampus R**	0.013 [0.040]	-0.066 to 0.093	0.735	0.013 [0.040]	-0.066 to 0.093	0.736
**dlPFC R**	**Thalamus L**	0.026 [0.038]	-0.050 to 0.103	0.490	0.015 [0.038]	-0.060 to 0.091	0.686
	**Thalamus R**	0.076 [0.044]	-0.013 to 0.165	0.091	0.068 [0.045]	-0.022 to 0.157	0.135
	**Caudate L**	0.060 [0.033]	-0.006 to 0.125	0.072	0.050 [0.033]	-0.015 to 0.115	0.133
	**Caudate R**	0.089 [0.031]	0.026 to 0.152	*0.006*	0.090 [0.032]	0.027 to 0.153	*0.006*
	**Hippocampus L**	0.016 [0.039]	-0.062 to 0.094	0.685	0.040 [0.038]	-0.036 to 0.115	0.295
	**Hippocampus R**	0.028 [0.041]	-0.053 to 0.109	0.487	0.047 [0.040]	-0.032 to 0.126	0.241

Based on 24 comparisons, with mean r = .227. between the outcomes, corrected α = .004. *Corrected for age, sex, education in years and framewise displacement. *Abbreviations:* dACC – dorsal anterior cingulate cortex; dlPFC – dorsolateral prefrontal cortex.

Table A.15 Nodal graph outcomes of sub-network key regions.

		Graph outcomes of selected network key regions					
		Crude model			Adjusted model[a]		
		B [SE]	95% CI	p-value	B [SE]	95% CI	p-value
**Participation coefficient**	**Dorsal anterior insula L**	-0.011 [0.010]	-0.031 to 0.009	0.283	-0.008 [0.010]	-0.028 to 0.012	0.412
	**Dorsal anterior cingulate cortex L**	-0.008 [0.010]	-0.028 to 0.011	0.388	-0.005 [0.010]	-0.025 to 0.015	0.607
	**Medial prefrontal cortex L**	-0.015 [0.011]	-0.038 to 0.008	0.189	-0.012 [0.011]	-0.034 to 0.011	0.299
	**Posterior cingulate cortex L**	-0.015 [0.010]	-0.035 to 0.005	0.147	-0.011 [0.010]	-0.032 to 0.009	0.272
	**Dorsal anterior cingulate cortex R**	-0.006 [0.009]	-0.024 to 0.012	0.520	-0.003 [0.009]	-0.022 to 0.016	0.749
	**Inferior parietal lobe R**	-0.010 [0.009]	-0.028 to 0.009	0.299	-0.005 [0.009]	-0.023 to 0.013	0.561
	**Posterior cingulate cortex R**	-0.014 [0.010]	-0.034 to 0.006	0.159	-0.010 [0.010]	-0.030 to 0.009	0.296
**Betweenness centrality[b]**	**Dorsal anterior cingulate cortex L**	0.366 [0.345]	-0.322 to 1.054	0.292	0.263 [0.339]	-0.414 to 0.939	0.441
	**Medial prefrontal cortex L**	0.158 [0.237]	-0.315 to 0.632	0.507	0.084 [0.233]	-0.381 to 0.548	0.721
	**Inferior parietal lobe R**	-0.031 [0.298]	-0.625 to 0.562	0.917	-0.182 [0.293]	-0.767 to 0.402	0.536
	**Dorsolateral prefrontal cortex R**	-0.041 [0.285]	-0.610 to 0.527	0.885	-0.056 [0.284]	-0.622 to 0.510	0.845
**Clustering coefficient**	**Dorsal anterior insula L**	0.001 [0.005]	-0.008 to 0.010	0.823	0.000 [0.005]	-0.009 to 0.010	0.967
	**Dorsal anterior cingulate cortex L**	0.000 [0.005]	-0.009 to 0.010	0.924	-0.001 [0.005]	-0.011 to 0.009	0.852
	**Inferior parietal lobe L**	-0.002 [0.005]	-0.011 to 0.008	0.724	-0.001 [0.005]	-0.011 to 0.008	0.778
	**Posterior cingulate cortex L**	0.001 [0.004]	-0.008 to 0.010	0.755	0.001 [0.005]	-0.008 to 0.010	0.860
	**Dorsal anterior insula R**	0.000 [0.005]	-0.009 to 0.010	0.924	0.001 [0.005]	-0.009 to 0.010	0.867
	**Dorsal anterior cingulate cortex R**	-0.001 [0.005]	-0.012 to 0.009	0.799	-0.002 [0.005]	-0.013 to 0.009	0.701
	**Dorsolateral prefrontal cortex R**	-0.003 [0.005]	-0.013 to 0.006	0.492	-0.004 [0.005]	-0.014 to 0.006	0.399
	**Medial prefrontal cortex R**	0.004 [0.005]	-0.006 to 0.013	0.429	0.003 [0.005]	-0.006 to 0.013	0.490
	**Posterior cingulate cortex R**	0.004 [0.005]	-0.006 to 0.014	0.392	0.004 [0.005]	-0.006 to 0.014	0.464

Correlation with behavioral outcomes at baseline informed selection of region for analysis. [a]Corrected for age, sex and education in years; [b]Statistics multiplied by 10^3 because of small values.

References

1. Esteban O, Markiewicz CJ, Blair RW, et al. fMRIPrep: a robust preprocessing pipeline for functional MRI. Nat Methods 2019; 16(1): 111-116.
2. Gorgolewski K, Burns CD, Madison C, et al. Nipype: a flexible, lightweight and extensible neuroimaging data processing framework in python. Front Neuroinform 2011; 5: 13.
3. Tustison NJ, Avants BB, Cook PA, et al. N4ITK: improved N3 bias correction. IEEE Trans Med Imaging 2010; 29(6): 1310-1320.
4. Avants BB, Epstein CL, Grossman M, Gee JC. Symmetric diffeomorphic image registration with cross-correlation: evaluating automated labeling of elderly and neurodegenerative brain. Med Image Anal 2008; 12(1): 26-41.
5. Zhang Y, Brady M, Smith S. Segmentation of brain MR images through a hidden Markov random field model and the expectation-maximization algorithm. IEEE Trans Med Imaging 2001; 20(1): 45-57.
6. Reuter M, Rosas HD, Fischl B. Highly accurate inverse consistent registration: a robust approach. Neuroimage 2010; 53(4): 1181-1196.
7. Dale AM, Fischl B, Sereno MI. Cortical surface-based analysis. I. Segmentation and surface reconstruction. Neuroimage 1999; 9(2): 179-194.
8. Klein A, Ghosh SS, Bao FS, et al. Mindboggling morphometry of human brains. PLoS Comput Biol 2017; 13(2): e1005350.
9. Evans AC, Janke AL, Collins DL, Baillet S. Brain templates and atlases. Neuroimage 2012; 62(2): 911-922.
10. Greve DN, Fischl B. Accurate and robust brain image alignment using boundary-based registration. Neuroimage 2009; 48(1): 63-72.
11. Jenkinson M, Bannister P, Brady M, Smith S. Improved optimization for the robust and accurate linear registration and motion correction of brain images. Neuroimage 2002; 17(2): 825-841.
12. Cox RW, Hyde JS. Software tools for analysis and visualization of fMRI data. NMR Biomed 1997; 10(4-5): 171-178.
13. Pruim RHR, Mennes M, van Rooij D, Llera A, Buitelaar JK, Beckmann CF. ICA-AROMA: A robust ICA-based strategy for removing motion artifacts from fMRI data. Neuroimage 2015; 112: 267-277.
14. Power JD, Mitra A, Laumann TO, Snyder AZ, Schlaggar BL, Petersen SE. Methods to detect, characterize, and remove motion artifact in resting state fMRI. Neuroimage 2014; 84: 320-341.
15. Behzadi Y, Restom K, Liau J, Liu TT. A component based noise correction method (CompCor) for BOLD and perfusion based fMRI. Neuroimage 2007; 37(1): 90-101.
16. Satterthwaite TD, Elliott MA, Gerraty RT, et al. An improved framework for confound regression and filtering for control of motion artifact in the preprocessing of resting-state functional connectivity data. Neuroimage 2013; 64: 240-256.
17. Lanczos C. Evaluation of noisy data. Journal of the Society for Industrial and Applied Mathematics, Series B: Numerical Analysis 1964; 1(1): 76-85.
18. Abraham A, Pedregosa F, Eickenberg M, et al. Machine learning for neuroimaging with scikit-learn. Front Neuroinform 2014; 8: 14.
19. van den Heuvel MP, Sporns O. Rich-club organization of the human connectome. J Neurosci 2011; 31(44): 15775-15786.
20. Baggio HC, Segura B, Junque C, de Reus MA, Sala-Llonch R, Van den Heuvel MP. Rich Club Organization and Cognitive Performance in Healthy Older Participants. J Cogn Neurosci 2015; 27(9): 1801-1810.
21. Bertolero MA, Yeo BTT, D'Esposito M. The diverse club. Nat Commun 2017; 8(1): 1277.
22. Alstott J, Panzarasa P, Rubinov M, Bullmore ET, Vertes PE. A unifying framework for measuring weighted rich clubs. Sci Rep 2014; 4: 7258.
23. Schaefer A, Kong R, Gordon EM, et al. Local-Global Parcellation of the Human Cerebral Cortex from Intrinsic Functional Connectivity MRI. Cereb Cortex 2018; 28(9): 3095-3114.
24. Kessels RPC, Bucks RS, Willison RW, Byrne LMT. [Location Learning Test: Manual]. 4th ed. Amsterdam: Hogrefe, 2016.

25. Nicholas LE, Brookshire RH, Maclennan DL, Schumacher JG, Porrazzo SA. Revised Administration and Scoring Procedures for the Boston Naming Test and Norms for Non-Brain-Damaged Adults. Aphasiology 1989; 3(6): 569-580.
26. Campo P, Morales M. Reliability and normative data for the Benton Visual Form Discrimination Test. Clin Neuropsychol 2003; 17(2): 220-225.
27. Schmand B, Houx P, de Koning I. [Norms neuropsychological assessments]. https://www.psynip.nl/en/2012.
